# Mavodelpar in patients with primary mitochondrial myopathy: a phase 1 trial

**DOI:** 10.1038/s41598-026-43287-0

**Published:** 2026-05-18

**Authors:** Renae J. Stefanetti, Chiara Pizzamiglio, Alasdair P. Blain, Oliver M. Russell, Lisa Alcock, Gavin Hudson, Jane Newman, Naomi Thomas, Charlotte Warren, Huizhong Su, Helen A. L. Tuppen, Philip Brown, David Houghton, Heather Hunter, Albert Z. Lim, Yi Shiau Ng, Catherine Feeney, Iwona Skorupinska, Louise Germain, Enrico Bugiardini, Michael G. Hanna, Robert McFarland, Robert D. S. Pitceathly, Gráinne S. Gorman

**Affiliations:** 1 Translational and Clinical Research Institute, Faculty of Medical Sciences, Newcastle University, Newcastle upon Tyne, NE2 4HH UK; 2 National Institute for Health and Care Research Newcastle Biomedical Research Centre, Newcastle upon Tyne, UK; 3 NHS Highly Specialised Service for Rare Mitochondrial Disorders, Newcastle upon Tyne Hospitals NHS Foundation Trust, Newcastle upon Tyne, UK; 4 Newcastle upon Tyne Hospitals NHS Foundation Trust, Newcastle upon Tyne, UK; 5 Department of Neuromuscular Diseases, University College London Queen Square Institute of Neurology, London, UK; 6 Queen Square Centre for Neuromuscular Diseases, NHS Highly Specialised Service for Rare Mitochondrial Disorders, The National Hospital for Neurology and Neurosurgery, London, UK; 7 Faculty of Medical Sciences, Newcastle University, Newcastle upon Tyne, UK

**Keywords:** Phase 1 trial, Rare disease, Mitochondrial disease, Mitochondrial myopathy, Peroxisome proliferator-activated receptor delta (PPARδ) agonist, Outcome measures, Diseases, Medical research

## Abstract

Primary mitochondrial myopathies (PMM) are rare, genetically-defined disorders characterised by defects of oxidative phosphorylation, predominantly affecting skeletal muscle. This Phase 1b open-label trial evaluated mavodelpar, a selective peroxisome proliferator-activated receptor delta (PPARδ) agonist, over 12 weeks (Part A), with an optional 36 week extension (Part B) in adults with PMM. The primary objective was to assess safety and tolerability, with secondary assessments of pharmacokinetics, pharmacodynamics, and exploratory performance, patient-reported, and muscle biopsy outcomes. Of the 23 participants who received mavodelpar, 17 completed Part A; none completed Part B due to premature study termination during the COVID-19 pandemic. Adverse events were mild-moderate severity, with headache and constipation most common (4/23 participants; 17.4% each). Exploratory measures showed a mean increase of 104 m in the twelve minute walk test (95% CI: 53 to 156) and a mean reduction of -10.5 points in patient-reported fatigue (95% CI: -16.3 to -4.6). No consistent changes in mitochondrial function were detected in muscle biopsies (n = 10), while transcriptomic profiling (n = 6) revealed modest upregulation of fatty acid–metabolism pathways. Although findings from this Phase 1b trial supported progression to later-phase evaluation, the subsequent Phase 2b trial did not demonstrate clinical efficacy for mavodelpar. The results reported here should be interpreted as exploratory and not indicative of therapeutic benefit. Nevertheless, this Phase 1b trial provides important methodological insights to inform future PMM clinical trial design and outcome measure development.

**Supplementary Information:**

The online version contains supplementary material available at 10.1038/s41598-026-43287-0, https://doi.org/10.25405/data.ncl.20747032.v1.

## Introduction

Primary mitochondrial diseases are a group of rare genetic disorders characterized by defects in oxidative phosphorylation (OXPHOS) and are considered the most prevalent group of inherited neurometabolic diseases^1^. Tissues with high-energy demands, such as muscle, brain and heart, are preferentially affected. Patients with Primary mitochondrial myopathies (PMMs) have pathogenic changes (e.g., depletion, deletions, or pathogenic point variants) in mitochondrial DNA (mtDNA) or nuclear DNA (nDNA), predominantly affecting skeletal muscle. Prominent symptoms of PMM include exercise intolerance, weakness, fatigue^2^, and pain^3^. Myopathy, which may be progressive, often impacts mobility and the ability to perform activities of daily living^4^. Although there has been an increase in the development of therapeutic investigations for PMM^5^, no effective treatments have been approved to date.

Mavodelpar is a selective peroxisome proliferator-activated receptor delta (PPARδ) agonist that has been shown to regulate the transcription of genes involved in fatty acid metabolism and glucose homeostasis^6^. PPARδ is highly expressed in metabolically active tissues, including liver, muscle, and adipose tissue, and is the most abundant PPAR isoform in skeletal muscle, where it is preferentially expressed in oxidative type I fibers^7^. Exercise training increases endogenous PPARδ expression in skeletal muscle^6^. In animal models, genetic overexpression and pharmacological activation of PPARδ increases the number of muscle fibres with high mitochondrial content, promoting fatty acid oxidization (FAO)^8^. Preclinical studies in Duchenne muscular dystrophy animal models have shown that PPARδ modulators upregulate mitochondrial regulatory pathways. These studies provide evidence that PPARδ agonism may be effective in ameliorating mitochondrial dysfunction^9,10^. The pan-PPAR agonist; bezafibrate, has also been shown to delay the formation of mtDNA deletions in the “mutator” mouse model of late onset myopathy^11^. While a small clinical trial showed benefits in patients with m.3243A>G mitochondrial disease, concerns were raised regarding its long-term safety^12^.

The rationale for a PPARδ-specific agonist treatment in patients with PMM is that it may ameliorate the cellular energy deficit by increasing FAO and OXPHOS activity. By increasing beta-oxidation of fatty acids, mavodelpar is postulated to reduce the reliance of OXPHOS on nicotinamide adenine dinucleotide dehydrogenase (NADH) oxidation by complex I. There is also evidence to suggest that PPARδ activation may increase mitochondrial biogenesis, however further investigation is warranted^13^. This Phase 1b trial was primarily designed to assess the safety and tolerability of mavodelpar in adults with PMM. In addition, it generates hypothesis-informing data spanning functional, patient-reported, pharmacodynamic and muscle molecular measures to inform outcome measure selection and guide interpretation for future PMM clinical trials. Although a subsequent later-phase evaluation has since been completed, the present report focuses on the Phase 1b findings and the methodological and biomarker insights they provide.

## Results

### Participants

**Fig 1:**
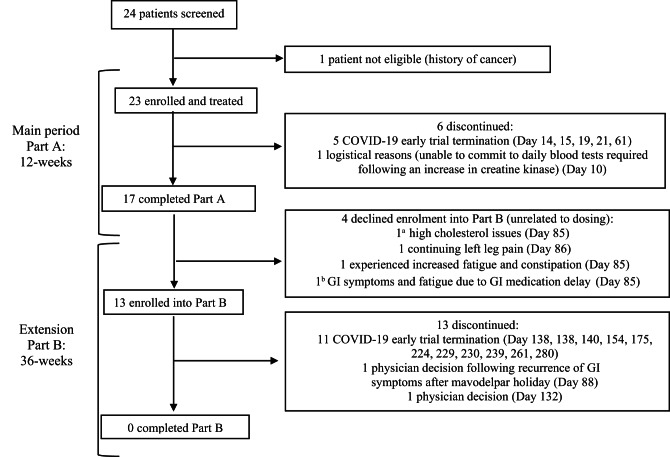
CONSORT Diagram. ^a^ Participant had ongoing hypercholesterolaemia along with additional cardiac risk factors, including smoking and diabetes mellitus. Due to a previous poor response to statins, bezafibrate (a disallowed concomitant hyperlipidaemia medication) was advised, which precluded continued participation into Part B. ^b^ The participant experienced a delay in obtaining their usual bowel medication, which consequently increased their constipation and fatigue. N.B. The Day denotes the withdrawal date, GI, gastrointestinal.

A total of 24 patients were screened for eligibility, of whom 23 were enrolled (Fig. 1). All 23 participants who received at least one dose of 100 mg mavodelpar constitute the Full Analysis Set (FAS). The first participant was enrolled on 10 May 2019. However, to manage the impact of COVID-19 and protect the interest of participants, the study was prematurely terminated on 31 May 2020. The Sponsor (Reneo Pharmaceuticals, Inc.) and the Principal Investigators made this decision due to the significant logistical challenges posed by the COVID-19 pandemic, as well as UK government guidance to protect patients with PMM, who were medically defined as extremely vulnerable to COVID-19. Although the early trial termination impacted the intended study period of 12 weeks in Part A and 48 weeks overall, the data obtained in Part A (first 12 weeks) provide important insights and are the focus on this manuscript. Interim safety data from Part B, collected up to early termination, are also reported to provide additional information on tolerability.

The mean age of participants was 54.5 (SD, 7.8) years. Fifteen participants (65.2%) were women, and 22 (95.7%) were of white race. Although the severity of clinical presentations varied among participants, myopathy was the dominant clinical feature (100% of participants had a Newcastle Mitochondrial Disease Adult Scale [NMDAS]^14^ subscale score ≥2), with exercise intolerance and gait impairment also frequently identified (91% of participants had a score >0 for both). Cardiac and respiratory involvement were infrequent, with 78.3% of participants scoring 0–1 on the respective NMDAS domains. Participant demographics and clinical characteristics are shown in Table Supplementary S1.

Six participants did not complete Part A: five participants were withdrawn for reasons related to COVID-19; and one participant withdrew consent at 10 days of treatment due to logistical reasons (inability to commit to daily blood tests required for monitoring following an increase in creatine kinase [CK]). Of the 17 participants who completed Part A, 13 entered Part B. However, no participants completed Part B due to early trial termination. As a result, Week 48 assessments were not available; longer-term functional, patient-reported, and disease-severity outcomes could therefore not be assessed. Most participants (n = 11) in Part B were withdrawn due to the COVID-19 pandemic, with the remaining two withdrawing due to the physician’s decision (Fig. 1). The mean duration of mavodelpar exposure was 18.9 weeks/131.5 days (range: 2–40 weeks/14–280 days).

### Safety and tolerability outcomes

The Full Analysis Set (FAS) and safety population included all 23 participants who received at least one dose of mavodelpar. In Part A, 85 Treatment-emergent adverse events (TEAEs) were reported by 20 of 23 participants (87.0%). There was one serious TEAE of hematoma, which was considered unrelated to mavodelpar, as it resulted from complications associated with the muscle biopsy performed at baseline. All TEAEs across the trial were classified as mild or moderate by the Investigator. There were no TEAEs that led to study discontinuation during Part A (Table 1).

**Table 1:** Summary of treatment-emergent adverse events – Part A.

	Mavodelpar 100 mg once daily			
	Full analysis set (N = 23)		Completers analysis set (N = 17)	
	Events	Participants, No. (%)	Events	Participants, No. (%)
Total number of TEAEs	85	20 (87·0)	69	16 (94·1)
Total number of TEAEs related to study drug^a^	55	13 (56·5)	41	10 (58·8)
Total number of serious TEAEs	1^b^	1 (4·3)^b^	1	1 (5·9)
Total number of serious treatment-related TEAEs^a^	0	0	0	0
Maximum severity^c^				
Mild	-	9 (39·1)	-	8 (47·1)
Moderate	-	11 (47·8)	-	8 (47·1)
Severe	-	0	-	0
Related TEAEs by maximum severity^a,c^				
Mild	-	5 (21·7)	-	5 (29·4)
Moderate	-	8 (34·8)	-	6 (35·3)
Severe	-	0	-	0
Relationship to study treatment^d^				
Not related	-	7 (30·4)	-	6 (35·3)
Possibly related	-	9 (39·1)	-	8 (47·1)
Probably related	-	4 (17·4)	-	2 (11·8)
Related	-	0	-	0
Total number of participants with TEAEs leading to study drug discontinuation	-	0	-	0
Total number of participants with TEAEs leading to death	-	0	-	0

Treatment-emergent adverse events (TEAEs) are defined as those adverse events that started after administration of mavodelpar or began prior to mavodelpar administration but increased in severity/relationship to mavodelpar after administration. ^a^Related TEAEs are those classified as possibly related, probably related or related. ^b^Event not related to study drug, related to post-procedural (baseline muscle biopsy) hematoma. ^c^If a participant experienced more than one TEAE, their most severe event is counted. ^d^If a participant experienced more than one TEAE, their event most related to study drug is counted.

The most frequent TEAEs in Part A reported by ≥ two participants (5%) categorised by System Organ Class (SOC) were: Gastrointestinal Disorders in nine participants (39.1%) (Table S2), Musculoskeletal and Connective Tissue Disorders in seven participants (30.4%), Nervous System Disorders in seven participants (30.4%), Skin and Subcutaneous Tissue Disorders in seven participants (30.4%), and Infections and Infestations in six participants (26.1%). The most frequently reported TEAEs by Preferred Term (PT) were constipation in four participants (17.4%), headache in four participants (17.4%), gastroesophageal reflux disease in three participants (13.0%), dry skin in three participants (13.0%), fatigue in three participants (13.0%), and neutropenia in three participants (13.0%). A summary of most frequent TEAEs in Part A is presented in Table2.

**Table 2:** Most frequent (≥2 participants (5 %)) treatment-emergent adverse events by System Organ Class and Preferred Term – Part A.

	Mavodelpar 100 mg once daily			
	Full analysis set (N = 23)		Completers analysis set (N = 17)	
System Organ Class Preferred Term	Events	Participants, No. (%)	Events	Participants, No. (%)
Gastrointestinal disorders	**19**^**a**^	**9 (39**·**1)**	**12**	**6 (35·3)**
Constipation	4	4 (17·4)	3	3 (17.6)
Gastroesophageal reflux	3	3 (13·0)	2	2 (11·8)
Diarrhea	2	2 (8·7)	1	1 (5·9)
Nausea	2	2 (8·7)	1	1 (5·9)
Musculoskeletal and Connective Tissue Disorders	**8**	**7 (30**·**4)**		
Muscle spasms	2	2 (8·7)	1	1 (5·9)
Pain in extremity	3	2 (8·7)	3	2 (11·8)
Nervous System Disorders	**9**	**7 (30**·**4)**		
Headache	4	4 (17·4)	3	3 (17.6)
Skin and Subcutaneous Tissue Disorders	**9**	**7 (30**·**4)**		
Dry skin	3	3 (13·0)	2	2 (11·8)
Infections and Infestations	**7**	**6 (26**·**1)**		
Viral infection	2	2 (8·7)	1	1 (5·9)
General Disorders and Administration Site Conditions	**6**	**5 (21**·**7)**		
Fatigue	3	3 (13·0)	3	3 (17.6)
Peripheral swelling	2	2 (8·7)	2	2 (11·8)
Investigations	**10**	**5 (21**·**7)**		
Alanine aminotransferase increased	2	2 (8·7)	1	1 (5·9)
Aspartate aminotransferase increased	2	2 (8·7)	1	1 (5·9)
Blood creatine kinase increased	2	2 (8·7)	1	1 (5·9)
Blood and lymphatic system disorders	**4**	**4 (17**·**4)**		
Neutropenia	3	3 (13·0)	3	3 (17·6)
Respiratory, thoracic and mediastinal disorders	**3**	**3 (13**·**0)**		
Oropharyngeal pain	2	2 (8·7)	2	2 (11·8)

Treatment-emergent adverse events (TEAEs) are defined as adverse events that started after administration of mavodelpar or began prior to mavodelpar administration but increased in severity/relationship to mavodelpar after administration. ^a^See Supplementary, Table S2 (TEAEs classified as Gastrointestinal Disorders).

**Fig 2:**
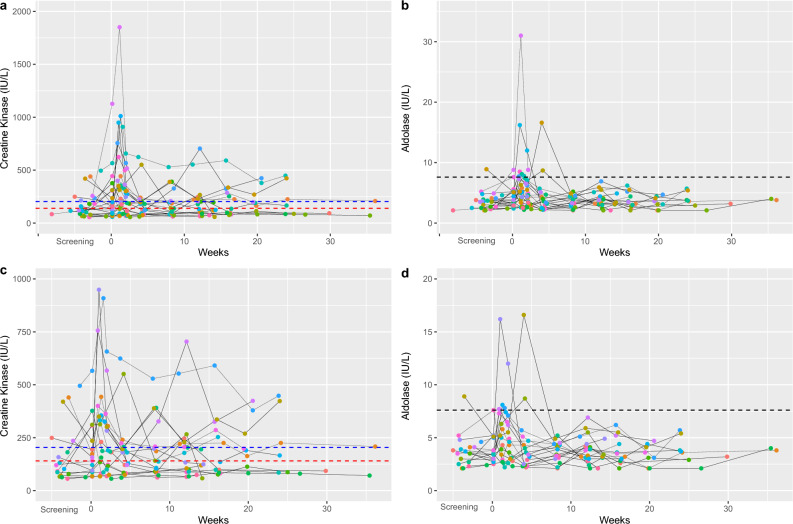
Evaluation of plasma biochemistry. Line plots of individual participants biochemistry data throughout the trial are presented. For each figure, plasma concentration (y-axis) is plotted against sampling times (x-axis). Individual colours represent individual participants to allow visualisation of biochemistry data over time; Week 0 represents the first dose of mavodelpar treatment (baseline). Graphs in the top panel (**a**, **b**) include the Full Analysis Set (FAS) population, while graphs in the bottom panel (**c**, **d**) represent the Completers Analysis Set population (Part A, i.e., 12 weeks). (**a**, **c**) Creatine kinase (CK) (IU/L) (n = 23 [FAS] (**a**) and n = 16 [Week 12] (**c**)). The CK (IU/L) normal concentration upper range for males (blue dashed line) and females (red dashed line) is plotted. (**b, d**) Aldolase (IU/L) (n = 22 [FAS] (**b**) and (n = 16 [Week 12] (**d**)). The normal concentration upper range is presented as a black dashed line.

There were 29 TEAEs with an onset during Part B, experienced by nine out of 13 participants (69.2%). The most frequently reported TEAEs by SOC with an onset in Part B were Infections and Infestations in six participants (46.2%) and Gastrointestinal Disorders in four participants (30.8%). The most frequently reported TEAEs in Part B were diarrhoea in two participants (15.4%) and migraine in two participants (15.4%). Leg pain in one participant led to a six-day temporary treatment discontinuation for further investigation (not dosed on Days 60–65). Although no cause was identified, the persistence of pain precluded continuation into Part B (participant-directed decision). One participant had a return of gastrointestinal symptoms after a treatment interruption of seven days, which led to study discontinuation in Part B (Table S3). There were no serious TEAEs reported in Part B, and no deaths occurred throughout the study. In both Parts A and B, no clinically meaningful changes were observed in vital signs, ECG parameters, or physical examination findings (Table S4). A summary of laboratory parameter data is provided in Table S5, with abnormalities of potential clinical importance (PCI) detailed in Table S6. Although several participants exhibited mild or transient CK fluctuations (Fig. 2a–c), such variation is recognised in PMM and did not meet protocol-defined criteria for clinical significance. Only two participants experienced CK elevations classified as PCI, both of which were transient and deemed non-serious. Increases in plasma aldolase (n = 8) were also transient and non-serious (Fig. 2b–d).

### Pharmacokinetics

Pharmacokinetic (PK) analyses were conducted on the PK population, which included all participants in the FAS who had at least one evaluable post-dose PK measurement. On Day 1, median mavodelpar plasma concentration increased up to the final sampling time of four hours, with a median concentration at four hours of 3870 ng/mL (range: 1300 ng/mL to 5820 ng/mL). As expected, mavodelpar concentration levels were below the limit of quantification prior to dosing. At Week 12, the median mavodelpar plasma concentration was 1060 ng/mL (n = 17), with concentrations increasing after dosing to a maximum median value of 4800 ng/mL at two hours, before reducing to 4040 ng/mL at four hours, consistent with expected accumulation from repeat administration. The median plasma concentration profiles were similar between participants with m.3243A>G and those with other pathogenic mtDNA defects.

### Pharmacodynamics

#### Serum biomarkers: FGF-21 and GDF-15

Pharmacodynamic samples for serum biomarkers (fibroblast growth factor 21 [FGF-21] and growth differentiation factor 15 [GDF-15]) were collected in Part A at baseline and Week 12. Concentrations of both FGF-21 and GDF-15 were highly variable. Baseline GDF-15 concentration means were similar between participants with m.3243A>G and those with other pathogenic mtDNA defects. While the mean baseline FGF-21 concentration was higher in participants with the m.3243A>G variant compared to those with other mtDNA defects, this difference was not statistically significant due to high interindividual variation (95% CI: −281, 1084; *p* = 0.22). There were small, non-significant changes in mean (SD) FGF-21 and GDF-15 concentrations from baseline to Week 12 (−10 ± 174 pg/mL [95% CI: −103, 82]; *p* = 0.81 and 141 ± 287 pg/mL [95% CI: −1, 294]; *p* = 0.14, respectively) in participants overall (Table S7). Mean GDF-15 and FGF-21 concentration changes from baseline were similar for the variant groups.

#### Acylcarnitine

Ten acylcarnitine species were measured at baseline and Week 12. While concentrations of carnitines were variable between participants, a significant decrease in isovaleryl-carnitine (C5) and a significant increase in propionyl-carnitine (C3) and octanoyl-carnitine (C8) were observed at Week 12 compared to baseline (*p* = 0.046 to 0.002) . Acetyl-carnitine (C2) was also increased, although this change was not statistically significant (Table S7).

#### Muscle biopsy analyses

Muscle biopsies were performed and analysed in 12 participants (Newcastle site only). Of these, two participants did not have a Week 12 post-treatment biopsy (one due to trial withdrawal and one due to biopsy complications at baseline, with a haematoma precluding a repeat biopsy). The average number of total fibres evaluated in muscle histopathology H&E sections was 302.5 ± 176.6. Analysis of mtDNA copy number, mtDNA heteroplasmy levels, and mitochondrial mass (porin) protein per fibre area revealed no significant changes after 12 weeks. Similarly, no notable average changes in mitochondrial function (OXPHOS protein levels, ATP/ADP ratio) from baseline to Week 12 were observed (Table S8). The lack of statistical difference is likely due to inconsistent responses between individuals (Table S9).

Carnitine palmitoyltransferase 1 B (CPT1B [mitochondrial carnitine transporter]) is a key enzyme responsible for the transport of fatty acids metabolites into mitochondria for β-oxidation^15^. To investigate whether mavodelpar treatment modulates fatty acid metabolism, CPT1B protein levels in skeletal muscle biopsies were measured. Levels were compared to unaffected controls, with the proportion of fibres exceeding the 95% predicted threshold (defined by controls)^16^ quantified. From baseline to Week 12, the proportion of fibres with higher than expected CPT1B expression increased (2.87 ± 16.66% fibres [95% CI: −9.05, 14.78]; *p* = 0.06), accompanied by a mean decrease in the proportion of fibres with lower than predicted CPT1B levels (−3.29 ± 4.82% fibres [95% CI: −674, 0·16]; *p* = 0.45) (Table S9). However, these changes showed considerable variability, with no consistent trends observed across participants.

Transcriptomic analysis in muscle biopsies at baseline and after 12 weeks of mavodelpar treatment (n = 6 paired samples; see Table S10) revealed ten significantly differentially expressed genes (false discovery rate < 0.05 and a log_2_ fold change < −0.58/>0.58, Fig. S1b, Table S11). Of these, six genes were increased, including two nuclear encoded mitochondrial proteins (*ABHD1*, abhydrolase Domain Containing 1 and *CPT1A*, carnitine palmitoyltransferase 1 A), and four were decreased, including one nuclear encoded mitochondrial protein (*MICU3*, mitochondrial calcium uptake family member 3) in samples at Week 12 compared to baseline (Table S11). To gain an overview of global alterations in gene expression patterns we performed gene set enrichment analysis (GSEA). After classifying into gene ontology terms, we identified 16 biological processes that were significantly enriched in Week 12 samples compared to baseline (adjusted *p* < 0.05, Table S12). Notably, gene pathways related to fatty acid metabolism (*CPT1A*), adipogenesis (*PREB*), P53 (*ST14*), and inflammatory response pathways (*AQP9* and *C5*) were all elevated in Week 12 mavodelpar treatment samples compared to baseline.

### Exploratory efficacy endpoints

#### Exercise/functional capacity

**Fig 3:**
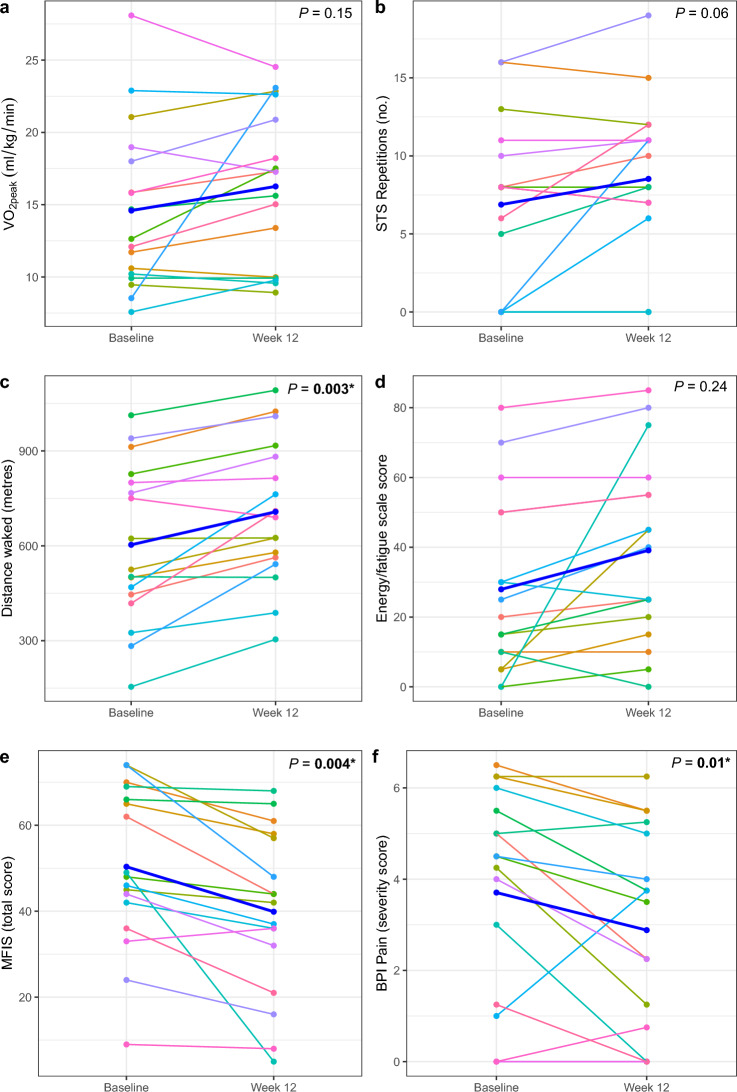
Exploratory efficacy outcomes with improvement. Spaghetti plots of individual participant response following 12 weeks of mavodelpar treatment (n = 17), for the following exploratory efficacy outcomes: (**a**) weight-adjusted peak oxygen consumption (VO_2_peak [ml/kg/min]) (**b**) sit-to-stand (STS) repetitions completed in 30 seconds; (**c**) distance walked in 12-minutes (12MWT); (**d**) energy/fatigue 36-Item Short Form Health Survey (SF-36) subscale score; (**e**) total Modified Fatigue Impact Scale (MFIS) score and (**f**) Brief Pain Inventory (BPI) - Short Form Pain Severity score. For all spaghetti plots, the group average is shown by the thickest royal blue line. *Statistically significant difference compared to Baseline (*p* < 0.05).

The mean increase in weight-adjusted peak oxygen consumption at Week 12 compared to baseline was not statistically significantly (1.67 ± 3.88 mL/kg/min [95% CI: −0.33, 3.66]; *p* = 0.15) (Table 3). While this corresponds to a small Cohen’s *d* effect size (0.2) equivalent to a minimal clinical important difference (MCID) established in similar populations^17^, there was considerable participant variability (Fig. 3a).

**Table 3:** Summary of efficacy exploratory endpoints.

	n	Full Analysis Set (N = 23)	n	Completers Analysis Set^a^ (N = 17)			
		Baseline		Baseline	Week 12	Change from Baseline	*P* value
		Mean ± SD		Mean ± SD	Mean ± SD	Mean ± SD (95% CI)	
Peak exercise test							
VO_2peak_ (ml/kg/min)	23	16·69 ± 7·06	17	14·59 ± 5·67	16·26 ± 5·35	1·67 ± 3·88 (−0·33 to 3·66)	0·15
Peak power (Watts)	23	83·6 ± 52·7	17	66·1 ± 24·7	74·4 ± 25·9	8·4 ± 14·3 (1·0 to 15·7)	0·07
Anaerobic threshold (ml/min)	23	686 ± 310	17	616 ± 214	682 ± 199	66 ± 206 (−40 to 172)	0·27
Lactate (mmol/L), end of test	23	5·37 ± 3·43	17	4·41 ± 3·09	5·31 ± 3·04	0·91 ± 1·80 (−0·02 to 1·83)	0·10
HR (bpm), end of test	23	120 ± 32	17	110 ± 29	127 ± 22	17 ± 19 (7 to 27)	**0·02**
RPE (6–20), end of test	23	16·57 ± 5·02	17	15·76 ± 5·55	18·06 ± 3·8	2·29 ± 4·69 (−0·12 to 4·70)	0·10
CWR sub-maximal exercise test^b,c^							
Duration before ceasing test (min:sec)^d^	19	21:06 ± 8:34	14	19:50 ± 8:42	23:43 ± 8:42	3:53 ± 5:31 (0:42 to 7:04)	**0**·**02**
Completed 30 minutes (No. participants)	19	7	14	4	8^e^	+4	NA
Lactate (mmol/L), end of test	18	4·12 ± 2·86	12	3·38 ± 2·59	3·60 ± 2·62	0·22 ± 1·30 (−0·61 to 1·04)	0·56
RPE (6–20), end of test	19	16·4 ± 3·4	14	17·0 ± 3·1	16·7 ± 3·1	−0·3 ± 2·8 (−1·9 to 1·3)	0·71
12-minute walk test							
Distance (m)	23	676 ± 258	17	603 ± 250	708 ± 226	104 ± 100 (53 to 156)	**0·003**
No. of Stops (No. participants)	23	28 (n = 8)	17	28 (n = 8)	19 (n = 5)	Stops: Decrease of 9^f^	0·38
30-second Sit-to-stand test							
Sit-to-stand (repetitions)^g^	23	8·2 ± 5·3	17	6·9 ± 5·5	8·5 ± 5·2	1·6 ± 3·30 (−0·05 to 3·3)	0·06
Unable to stand (No. participants)	23	5	17	5	3	−2	NA
Disease severity – NMDAS^h^							
Total score (0–140)	23	23·4 ± 9·8	17	25·6 ± 10·2	25·5 ± 12·0	−0·1 ± 5·2 (−2·8 to 2·6)	0·96
Perceived QoL – SF-36, v1 items^i^							
Physical functioning	23	45·9 ± 29·6	17	36·8 ± 27·4	44·1 ± 28·3	7·4 ± 18·9 (−2·4 to 17·1)	0·52
Role limitations (physical health)	23	41·3 ± 35·0	17	39·7 ± 39·6	38·2 ± 43·4	−1·5 ± 27·2 (−15·5 to 12·5)	0·86
Role limitations (emotional problems)	23	73·9 ± 36·2	17	70·6 ± 40·6	64·7 ± 41·6	−5·9 ± 47·5 (−30·3 to 18·5)	0·85
Energy/Fatigue	23	32·6 ± 23·9	17	27·9 ± 25·2	39·1 ± 26·4	11·2 ± 19·6 (1·1 to 21·2)	0·24
Emotional well-being	23	72·9 ± 18·7	17	72·5 ± 19·1	70·4 ± 20·9	−2·1 ± 9·8 (−7·2 to 2·9)	0·78
Social functioning	23	64·1 ± 22·7	17	61·0 ± 23·3	61·8 ± 26·7	0·7 ± 16·8 (−7·9 to 9·4)	0·86
Pain	23	58·4 ± 30·9	17	49·4 ± 29·6	55·3 ± 24·9	5·9 ± 23·6 (−6·2 to 18·0)	0·78
General health	23	40·9 ± 21·5	17	38·8 ± 23·6	40·3 ± 20·4	1·5 ± 12·8 (−5·1 to 8·1)	0·85
Perceived fatigue impact – MFIS							
MFIS total score (0–84)^j^	23	46·5 ± 17·5	17	50·4 ± 18·6	39·9 ± 19·1	−10·5 ± 11·4 (−16·3 to −4·6)	**0·004**
Perceived pain – BPI – (short form)							
Pain Severity (0–10)^k^	22	3·15 ± 2·39	17	3·72 ± 2·37	2·91 ± 2·23	−0·81 ±1·46 (−1·56 to −0·06)	**0·01**
Pain Interference (0–10)^l^	22	2·53 ± 2·71	17	3·18 ± 2·75	2·92 ± 2·57	−0·26 ± 1·63 (−1·10 to 0·58)	0·96

CWR, constant work rate sub-maximal exercise test; NMDAS, Newcastle Mitochondrial Disease Adult Scale; RPE, Rate of Perceived Exertion. ^a^N.B: Post-hoc analyses (after database lock), not specified in the statistical analysis plan, have been included: Lactate, HR, RPE (Peak exercise test); No. of participants who completed 30 minutes (CWR sub-maximal exercise test); no. of stops (12MWT). Completers Analysis Set: those participants who completed Part A; the 12 week treatment phase. Only those participants with data at both baseline and Week 12 are included in summaries. ^b^Three participants were excluded from the analysis due to a deviation from protocol (test conducted with workload < 50% of peak exercise capacity). One participant was unable to conduct the test due to a drop in blood pressure. ^c^N.B: Data analysis changed (after database lock), from only including data from participants who reached exhaustion (i.e., ceased cycling) prior to 30 minutes (maximum duration of the test), to now include data from all participants who either completed 30 minutes or who exhausted/ceased cycling prior to 30 minutes. Endpoints were measured immediately upon test cessation. ^d^Following 12 weeks, of the nine participants who had an increase in exercise duration – five had improvement (decrease) in either HR or RPE. Of the four participants who maintained the maximal exercise duration of the test (30 mins) at baseline and Week 12, three had improved (decrease) HR or RPE. ^e^All participants who exercised for 30 minutes at baseline (maximum duration of test) also exercised for 30 minutes at Week 12. ^f^Three participants who stopped at baseline did not stop during the 12 week 12MWT. Improvement (i.e., decreased in the number of stops at 12 weeks) occurred in two participants (one participant stopped six times at baseline and four times during the Week 12 assessment; a second participant stopped four times at baseline and three times at the Week 12 assessment). Worse performance (i.e., increase in the number of stops at Week 12) occurred in one participant (three stops at baseline, four stops at Week 12). No change in performance (i.e., number of stops at baseline and Week 12 was the same) in two participants (each stopping six and two times at both timepoints). ^g^Includes participants who were unable to complete the STS test i.e., scored 0. Two participants unable to stand at baseline completed six and 11 repetitions at Week 12. ^h^Total NMDAS score is calculated by summing the scores obtained for each section (section I to III). Higher scores indicate more severe disease. ^i^Item scores are summed and transformed into a scale score, (0–100). The higher score defines a more favourable health state. ^j^Total MFIS score is computed by adding scores on the physical (9-items), cognitive (10-items), and psychosocial (2-items) subscales. Higher scores indicate a greater impact of fatigue on a person’s activities. ^k^BPI – (Short Form) Pain Severity score is the average of the four pain items (a mean severity score). 0=no pain and 10=pain as bad as you can imagine. ^l^BPI – (Short Form) Pain Interference score (impact of pain on functioning) is scored as the mean of the 7 interference items. 0=no interference and 10=interferes completely. Significant values are in bold.

The constant work rate (CWR) sub-maximal exercise test was performed on a recumbent bike for 30 minutes (or until exhaustion) at 50–60% of the participant’s maximal capacity. Fourteen participants completed the test at baseline and Week 12 at the same constant workload. At baseline, four participants completed the full 30 minutes of CWR exercise, compared to a total of eight participants at Week 12. The mean (SD) significant increase in exercise duration after 12 weeks was 3:53 ± 5:31 min:sec (95% CI: 0:42, 7:04; *p* = 0.02) (Table 2); however, this is likely an underestimate due to the maximum duration being capped at 30 minutes.

For the 30-second sit-to-stand assessment (n = 17), changes from baseline to Week 12 ranged between −1 to 11 repetitions. Seven participants (41%) improved, six had no change, and four showed a decrease in the number of repetitions compared to baseline. The mean (SD) baseline value was 6.9 ± 5.5 sit-to-stands (range: 0 to 16), which increased, although non-significantly, to a mean (SD) of 8.5 ± 5.2 at Week 12 (mean increase: 1.6 ± 3.3 sit-to-stands [95% CI: −0.05, 3.3]; *p* = 0.06) (Table 2, Fig. 3b). This response was considered clinically non-significant, as the mean increase of 1.6 repetitions is below the 2-repetition MCID previously established^18^.

The mean (SD) significant increase in 12MWT distance at Week 12 compared to baseline was 104 ± 100 meters (95% CI: 53, 156; *p* = 0.003), equating to a relative average improvement of 26.4% (range: −8.0 to 97.4%). An improvement was observed in 15/17 (88%) of participants (Fig. 3c), with 13 of these 15 participants increasing their 12MWT distance by more than 60 meters, estimated to represent the MCID based on Cohen’s D standardised measure of change^19^.

#### Disease severity and patient-reported outcomes

There was no appreciable change in disease severity as measured by the NMDAS (*p* = 0.96). The mean (SD) NMDAS total disease scores (range: 0–140) were similar at baseline (25.6 ± 10.2) and Week 12 (25.5 ± 12.0) (Table 3).

Although not statistically significant, a mean change of at least four points from baseline to Week 12 was observed for certain subscale items of the SF-36 (score range: 0–100), indicating an improvement in perceived health. Thirteen of the 17 participants (76.5%) showed an improvement in their energy/fatigue. Other scales with notable changes included physical functioning (mean increase of 7.4 ± 18.9), pain (mean increase of 5.9 ± 23.6), and role limitations due to emotional problems (mean reduction/worsening of 5.9 ± 47.5) (Table 3). The total SF-36 mean (SD) increase of 11.2 ± 19.6 (95% CI: 1.1, 21.2) observed at Week 12 was not statistically significant (*p* = 0.24) (Table 3, Fig. 3d). However, the improvement exceeded the typical MCID^20^, suggesting a meaningful improvement in perceived QoL.

Participants perceived a statistically significant improvement in fatigue, with a mean decrease in MFIS total score from baseline to Week 12 (−10.5 ± 11.40 [95% CI: −16.3, −4.6], *p* = 0.004) (Table 3), indicating a clinically meaningful improvement^21^. At Week 12, 94% (n = 16/17) of participants reported an improvement in fatigue (Fig. 3e). Significant improvements were also observed in the physical (*p* = 0.008) and cognitive (*p* = 0.004) MFIS subscales at Week 12 compared to baseline (Table S13).

For the Brief Pain Inventory (n = 17), baseline pain severity (3.72 ± 2.37) and pain interference (3.18 ± 2.75) scores were low. At Week 12, a small but statistically significant reduction in pain severity was observed (−0.81 ± 1.46 [95% CI: −1.56, −0.06]; *p* = 0.01) (Fig. 3f). However, pain interference (reflecting the functional impact of pain in daily life), did not significantly change (−0.26 ± 1.63 [95% CI: −1.10, 0.58];* p* =0.96) (Table 3, Table S14). Neither measure met the typical MCID for clinically meaningful improvement^22^.

## Discussion

This Phase 1b study evaluated the safety and tolerability of mavodelpar in adult patients with mtDNA-related PMM. Overall, mavodelpar was generally well tolerated, with all TEAEs classified as mild or moderate in severity and no treatment-related serious adverse events. Some participants experienced transient elevations in serum CK and aldolase levels (non-specific markers of muscle damage), predominantly at Week 1. The cause of this increase is unclear but may reflect muscle damage from the baseline maximal exercise test^23^; all resolved without intervention or interruption of the study drug.

Total distance achieved during the 12MWT demonstrated the largest mean change among the exploratory outcome measures. Previous drug therapy trials in patients with PMM have frequently used the six-minute walk test (6MWT) but have failed to demonstrate efficacy as either a primary^24^ or secondary^25^ endpoint. Ceiling effects of the 6MWT have also been observed in other patient groups, particularly those with milder phenotypes^26^. This trial is the first to utilise the 12MWT in a PMM interventional study. Although the majority of participants (15/17) showed improvement, further validation in larger cohorts is required before its responsiveness and suitability as an efficacy endpoint can be generalised. Our findings indicate that the 12MWT is a safe and feasible assessment in an ambulatory PMM population and supports its further evaluation as a potential outcome measure in future clinical trials. However, given the open-label design and lack of a control group, these improvements should be interpreted as exploratory. Further validation of the 12MWT, including responsiveness and clinically meaningful change thresholds, is required in larger PMM cohorts.

Exploratory skeletal muscle biopsy analysis after 12 weeks of mavodelpar revealed an increase in CPT1B protein content above wild-type predictive intervals in over half of all biopsied participants, suggesting a possible biological response in a subset of patients. However, CPT1B transcript levels were unchanged. Transcriptomic analysis demonstrated upregulation of pathways involved in inflammation (complement, JAK/Stat signalling) and mitochondrial function, including fatty acid metabolism, MTORC1, and P53 signalling. Interestingly, *CPT1A* expression increased significantly, while *CPT1B* was unchanged. CPT1A is the dominant isoform in liver and fibroblasts, whereas CPT1B is dominant in heart and skeletal muscle. CPT1A is a rate limiting enzyme in mitochondrial β-oxidation and is regulated by PPARδ^27,28^, although it is not a direct target of mavodelpar. The absence of *CPT1B* upregulation may reflect its already high baseline abundance (5,070 vs. 970 for *CPT1A)*, potentially limiting further induction. Divergent CPT1A/B responses could alternatively be explained by off-target effects or compensatory responses, underlying tissue heterogeneity, or inherent variability in biopsy sampling. Given the small sample size (n = 12 for protein; n = 6 for RNA-sequencing), these findings should be interpreted cautiously.

Mechanistically, CPT1 (A/B) catalyses the conversion of cytosolic long-chain acyl-CoA into acylcarnitine at the outer mitochondrial membrane, enabling import into the mitochondrial matrix via the carnitine shuttle (carnitine–acylcarnitine translocase and carnitine palmitoyltransferase [CPT2]) for β-oxidation and generation of acetyl-CoA to fuel the TCA cycle and OXPHOS^29,30^. Therefore, increased CPT1 expression could facilitate mitochondrial long-chain fatty acid entry and increase the capacity for β-oxidation. Plasma acylcarnitine’s provide systemic biochemical context but may not directly reflect skeletal muscle CPT1-dependent long-chain FAO. Plasma pharmacodynamic acylcarnitine profiling at Week 12 showed statistically significant changes confined to propionyl-carnitine (C3; increased), isovaleryl-carnitine (C5; decreased) and octanoyl-carnitine (C8; increased), with no significant changes in acetyl-carnitine (C2) and free carnitine, or the long-chain species measured (tetradecenyl-carnitine, C14; palmitoyl-carnitine, C16). As C3/C5 are not specific to long-chain FAO and C8 is CPT1-independent, these findings are supportive but non-specific biochemical context with respect to CPT1-dependent long-chain FAO. Taken together, these exploratory findings are consistent with a shift toward a more oxidative metabolic phenotype following mavodelpar treatment. However, alternative explanations, such as differences in training status^31,32^, nutritional intake, or disease-related alterations in mitochondrial or peroxisomal function^33,34^ cannot be excluded, Given these uncertainties and the small sample size, these biopsy-based findings should be interpreted cautiously and viewed as preliminary insights into potential biological activity rather than evidence of a definitive therapeutic effect.

Despite improvements in the 12MWT, no significant group-level changes were detected in mitochondrial mass, OXPHOS subunit levels, or the ATP/ADP ratio after 12 weeks of treatment. This mismatch between functional improvements and largely unchanged biochemical measures may indicate that improvements in walking distance reflect unmeasured physiological adaptations, such as enhanced coordination, fatigue resistance, or vascular changes. Alternatively, it may reflect the small samples size, limitations in the sensitivity of the biopsy-based assays used, or placebo or behavioural effects, including increased motivation or effort. However, the absence of change in participants’ perception of exertion (Borg scale), or heart rate response suggests that increased effort alone is unlikely to account for the 12MWT improvement. Overall, these findings underscore that the exploratory FAO-related signals should be interpreted cautiously and do not provide evidence of a consistent mitochondrial effect of mavodelpar.

Exploratory self-reported outcomes suggested potential improvements in fatigue and pain severity with mavodelpar. Fatigue is highly prevalent in PMM^35,36^ and has been identified as a top-ten research priority by patients, caregivers, and healthcare professionals (n = 147)^37^. Although this trial is among the first in PMM to report a decrease in self-reported fatigue following treatment, interpretation is limited by the open-label design and absence of a placebo comparator. Placebo effects in self-reported outcomes, particularly fatigue and pain, are well recognised, including in neuromuscular disease trials^38,39^, and may therefore contribute to the observed changes. Nevertheless, the magnitude of improvement in MFIS scores exceeded thresholds typically considered clinically meaningful^40^, indicating that patient-reported signals merit cautious interpretation and further validation in future studies.

Pain is also a common symptom in patients with mtDNA variants, particularly for patients with a myopathic phenotype^41^. Importantly, mavodelpar treatment did not worsen patient-reported pain severity or its impact on daily activities. A small but statistically significant decrease in the mean BPI severity score was observed at Week 12 compared to baseline; however, the improvement in item scores were less than the two-point minimum, typically considered clinically significant in other disease cohorts^42^.

Study limitations included potential biases related to study design (e.g., open label, absence of a control/placebo comparator), potential subjective bias in patient-reported outcomes, and performance bias (e.g., a learning effect) in functional/exercise assessments. Although participants were instructed not to change their exercise regime, habitual physical activity was not objectively monitored, limiting control for behavioural confounders. This Phase 1b study was not designed or powered to evaluate efficacy, and the lack of established PMM-specific MCID values for most endpoints limits interpretation. In addition, the 12 week treatment period in Part A is insufficient to evaluate the persistence of functional changes or longer-term impact on disease course. Premature termination of Part B precluded completion of the planned 48 week follow-up, limiting assessment of longer-term (48 week) safety and the completion of pre-specified longer-term functional, patient-reported, and disease severity assessments. The large number of exploratory endpoints without adjustments for multiplicity increases the risk of false-positive findings. Only a subset of participants had paired biopsies (n = 10), and inherent variability in biopsy sampling limits sensitivity to detect subtle changes. As muscle fibre type was not assessed, we cannot determine whether changes in endurance capacity (12MWT distance) reflect fiber-type adaptations. ATP/ADP ratios were measured at rest, which may not reflect a mitochondrial defect, as previous studies have indicated slower ATP synthesis recovery rates in PMM after acute exercise^43^. Finally, study modifications due to the COVID-19 pandemic, such as increased remote site monitoring in place of on-site visits, impacted verification of compliance data.

Despite these limitations, the Phase 1b trial met its primary objective, demonstrating that mavodelpar was generally well tolerated in adults with PMM. Exploratory analyses also suggested potential improvements across several functional and patient-reported measures, most notably the 12MWT and MFIS fatigue scores, informing the design of the subsequent Phase 2b study. These exploratory findings, which should be interpreted as hypothesis generating, rather than evidence of efficacy, supported further clinical evaluation of mavodelpar. However, the Phase 2b trial (ClinicalTrials.gov identifier: NCT03862846) did not meet its primary endpoint and was discontinued. The results of the Phase 2b trial will be reported separately; however, the reasons for this discrepancy may reflect differences in trial duration and patient population, as well as the possibility that the exploratory findings in this early-phase study were influenced by limited statistical power, biological variability, or placebo effects. This outcome highlights the well-recognised challenge in mitochondrial disease drug development, where promising early-phase findings frequently fail to translate into demonstrated clinical efficacy in more rigorous later-phase trials^44^.

These findings reinforce the need for future clinical trials in PMM to incorporate larger sample sizes where feasible, alongside innovative and appropriate trial designs tailored to rare diseases, such as adaptive or Bayesian approaches, which can improve efficiency and inference in small, heterogeneous populations^45^. Refinement and validation of sensitive functional endpoints, such as the 12MWT, will be essential to ensure their reproducibility and clinical relevance across diverse PMM populations. Equally important will be the rigorous validation of surrogate biomarkers, integration of patient-defined meaningful outcomes, and the use of digital health technologies and decentralised trial methods to enable real-world assessment and overcome the practical limitations of small, geographically dispersed cohorts. Equally important is the validation of surrogate biomarkers, the integration of patient priorities when defining meaningful outcomes, and the use of digital health tools and decentralised trial methods to capture real-world functional performance. Collectively, these strategies may help to mitigate the methodological challenges inherently posed by clinical trials in small, clinically heterogeneous PMM populations. Importantly, early-phase efficacy signals must be interpreted with caution, as illustrated by the lack of efficacy observed in the subsequent Phase 2b trial.

## Methods

### Study design

The study was a Phase 1b open-label, two-site trial to evaluate the safety, tolerability, pharmacokinetics, pharmacodynamics, and exploratory efficacy of mavodelpar in patients with PMM caused by mtDNA defects. This study was conducted in two parts, Part A and Part B. During Part A, participants received 100 mg mavodelpar orally once daily for 12 weeks (Study design schematic/outcome measurement schedule available in Fig. S2 and Table S15). The 100 mg once daily dose was selected based on prior Phase 1 studies in healthy volunteers and individuals with dyslipidaemia; the full dose selection rationale is provided in the Clinical Study Protocol, Section 1.5 (Extended Data). Participants who tolerated mavodelpar in Part A had the option to extend dosing for an additional 36 weeks in Part B. However, the study was prematurely terminated during the public health emergency arising from the COVID-19 pandemic to ensure the safety of trial participants. Participants were enrolled from two study sites in the United Kingdom: the Newcastle upon Tyne Hospitals NHS Foundation Trust, Newcastle upon Tyne, and University College London Hospitals NHS Foundation Trust, London. The study was performed in accordance with local laws, the International Conference on Harmonization Good Clinical Practice guidelines, and the Declaration of Helsinki. As a Phase 1b clinical trial of an investigational medicinal product, regulatory authorisation was obtained from the Medicines and Healthcare products Regulatory Agency (MHRA), and a favourable ethical opinion was granted by the North East – Newcastle and North Tyneside 1 Research Ethics Committee (Ref: 18/NE/0309). The trial was registered with ClinicalTrials.gov (01/03/2019, NCT03862846) and the EU Clinical Trials Register (2018–002969-19). Participants continued standard clinical care throughout the study. Concomitant medications were permitted unless explicitly prohibited in the protocol, and required washout was completed prior to baseline dosing (Table S16). No formal patient or public involvement occurred in the design or conduct, of this trial.

### Participants

The study enrolled men and women (aged ≥18 years) with a confirmed genetic diagnosis of mitochondrial disease, including m.3243A>G (NC_012920.1) or other rarer pathogenic mtDNA variants, and clinical evidence of PMM according to the 2016 Rome Consensus recommendations^46^. The full eligibility criteria are provided in the Supplementary File (Study Protocol Summary, 1·1·1). All participants provided written informed consent prior to study entry.

### Outcome measurements

The primary objective was the assessment of safety and tolerability of mavodelpar treatment, evaluated by the incidence and severity of adverse events during the initial 12-week treatment period (Part A). Treatment-emergent adverse events (TEAEs) were classified according to the Medical Dictionary for Regulatory Activities (MedDRA) System Organ Class (SOC) and Preferred Terms version 21.1. The total number of TEAEs and treatment-emergent serious adverse events (TESAEs), as well as those considered related to the study treatment by the Investigator, were tabulated. The severity of TEAEs (mild, moderate, or severe) was assessed by Investigators and categorised based on pre-specified criteria (see Clinical Study Protocol, available in Extended Data).

Secondary safety endpoints were defined as changes from baseline and the incidence of clinically significant changes in laboratory safety tests (haematology and biochemistry), ECG parameters, and supine vital signs (blood pressure, pulse rate, and body temperature).

Pharmacokinetic (PK) and pharmacodynamic evaluations were additional endpoints. Blood samples for PK analysis were collected at pre-dose and 1, 2 and 4 hours post-dose on Day 1 of dosing and at Week 12, and post-treatment at Weeks 1, 2, 4, and 8. Pharmacodynamic analyses included effects of mavodelpar on serum biomarkers (fibroblast growth factor 21 [FGF-21] and growth/differentiation factor 15 [GDF-15]), acylcarnitine profile, and muscle histopathology analysis conducted on biopsies specimens performed at baseline and after 12 weeks. Details procedures are provided in the Supplementary File (Study Protocol Summary, 1·1·2).

Predefined exploratory efficacy evaluations were categorised into (i) exercise capacity as measured by peak and constant work rate (CWR) sub-maximal exercise tests; (ii) functional assessments as measured by the 12-minute walk test (12MWT) and 30 second sit-to-stand (STS); (iii) disease severity as quantified by NMDAS^14^; (iv) quality of life evaluated using version 1 of the RAND 36-Item Short Form Health Survey (SF-36); (v) perceived fatigue impact gauged by the Modified Fatigue Impact Scale (MFIS); (vi) pain experience and pain interference rated on the Brief Pain Inventory (BPI) - Short Forms, and (vii) muscle biopsy analyses, including assessment of mtDNA copy number and mtDNA heteroplasmy in muscle, OXPHOS protein abundance (complex I and complex IV) via quadruple immunofluorescence, CPT1B protein levels quantified by immunofluorescence, skeletal muscle ATP/ADP ratio measured via luminescence and, where sufficient biopsy material remained, transcriptomic analysis.

The 12MWT was selected over the commonly used six-minute walk test (6MWT) because its longer duration increases reliance on fatty acid oxidation, aligning with the proposed mechanism of mavodelpar. The 12MWT has also been shown to closely correlate with changes in VO₂max and may provide greater sensitivity for detecting changes in endurance^47^_._

### Statistical analysis

The planned target sample size of 24 participants (12 participants with m.3243A>G and 12 with rarer pathogenic mtDNA variants) was empirically determined to meet the study objectives. No formal hypothesis testing was planned. The Full Analysis Set (FAS) and safety population for analysis included all participants who received ≥1 dose of mavodelpar. Efficacy analyses were exploratory in nature and were conducted in patients who received ≥1 dose of mavodelpar; however, following the termination of the study due to the COVID-19 pandemic the primary population for the assessment of efficacy was based on those participants who completed the Week 12 visit in Part A (Week 12 Completers Analysis Set population). Changes from baseline were summarised using descriptive statistics for continuous data (e.g., mean, SD) and frequency tables (counts and percentages) for categorical data. Subgroup analyses (m.3243A>G versus other pathogenic mtDNA variants) showed no clinically relevant differences (data not shown). The Statistical Analysis Plan is provided in the Extended Data, with additional post hoc analyses and measures of MCID noted where applicable. No formal hypothesis testing was planned in the Statistical Analysis Plan. Exploratory hypothesis tests were conducted for exploratory endpoints to aid interpretation. Between-group comparisons were evaluated using independent-samples t-tests. Resulting p-values are presented for descriptive purposes only and should not be interpreted as confirmatory, as the study was not powered for formal inference and no adjustments for multiple testing were applied.

## Supplementary Information

Supplementary Information.

## Data Availability

*Extended Data* - The anonymised individual participant data is publicly available in Figshare 10.25405/data.ncl.20747032^48^. The Clinical Study Protocol and Statistical Analysis Plan, including all amendments, are also available in Figshare^48^. RNA sequencing data is deposited into the NCBI sequence read archive under accession number: PRJNA1044583 - [ID 1044583 - BioProject - NCBI] ( https://www.ncbi.nlm.nih.gov/bioproject/PRJNA1044583). Data will be retained for the lifetime of the repository.

## References

[1] Gorman, G. S. et al. Prevalence of nuclear and mitochondrial DNA mutations related to adult mitochondrial disease. *Ann. Neurol.* **77**, 753–759. 10.1002/ana.24362 (2015). 25652200 10.1002/ana.24362 PMC4737121

[2] Karaa, A. et al. Characterization of fatigue in primary mitochondrial myopathies. *Neurol. Clin. Pract.* **14**, 200229. 10.1212/CPJ.0000000000200229 (2024). 10.1212/CPJ.0000000000200229 PMC10790285 38229875

[3] van den Ameele, J. et al. Chronic pain is common in mitochondrial disease. *Neuromuscul. Disord.* **30**, 413–419. 10.1016/j.nmd.2020.02.017 (2020). 32334903 10.1016/j.nmd.2020.02.017 PMC7306151

[4] Taivassalo, T. & Haller, R. G. Implications of exercise training in mtDNA defects—Use it or lose it. *Biochimica et Biophysica Acta (BBA) - Bioenergetics* **1659**, 221–231 (2004). 15576055 10.1016/j.bbabio.2004.09.007

[5] Tinker, R. J., Lim, A. Z., Stefanetti, R. J. & McFarland, R. Current and emerging clinical treatment in mitochondrial disease. *Mol. Diagn. Ther.* **25**, 181–206. 10.1007/s40291-020-00510-6 (2021). 33646563 10.1007/s40291-020-00510-6 PMC7919238

[6] Wang, Y. X. et al. Regulation of muscle fiber type and running endurance by PPARdelta. *PLoS Biol.* **2**, e294. 10.1371/journal.pbio.0020294 (2004). 15328533 10.1371/journal.pbio.0020294 PMC509410

[7] Oliver, W. R. Jr. et al. A selective peroxisome proliferator-activated receptor δ agonist promotes reverse cholesterol transport. *Proc. Natl. Acad. Sci. U. S. A.* **98**, 5306–5311. 10.1073/pnas.091021198 (2001). 11309497 10.1073/pnas.091021198 PMC33205

[8] Wang, Y. X. PPARs: Diverse regulators in energy metabolism and metabolic diseases. *Cell Res.* **20**, 124–137. 10.1038/cr.2010.13 (2010). 20101262 10.1038/cr.2010.13 PMC4084607

[9] Bell, E. L. et al. PPARδ modulation rescues mitochondrial fatty acid oxidation defects in the mdx model of muscular dystrophy. *Mitochondrion* **46**, 51–58. 10.1016/j.mito.2018.02.006 (2019). 29458111 10.1016/j.mito.2018.02.006

[10] Narkar, V. A. et al. AMPK and PPARdelta agonists are exercise mimetics. *Cell* **134**, 405–415. 10.1016/j.cell.2008.06.051 (2008). 18674809 10.1016/j.cell.2008.06.051 PMC2706130

[11] Yatsuga, S. & Suomalainen, A. Effect of bezafibrate treatment on late-onset mitochondrial myopathy in mice. *Hum. Mol. Genet.* **21**, 526–535. 10.1093/hmg/ddr482 (2012). 22012983 10.1093/hmg/ddr482

[12] Steele, H. et al. Metabolic effects of bezafibrate in mitochondrial disease. *EMBO Mol. Med.* **12**, 11589. 10.15252/emmm.201911589 (2020). 10.15252/emmm.201911589 PMC7059007 32107855

[13] Kleiner, S. et al. PPAR{delta} agonism activates fatty acid oxidation via PGC-1{alpha} but does not increase mitochondrial gene expression and function. *J. Biol. Chem.* **284**, 18624–18633. 10.1074/jbc.M109.008797 (2009). 19435887 10.1074/jbc.M109.008797 PMC2707195

[14] Schaefer, A. M. et al. Mitochondrial disease in adults: A scale to monitor progression and treatment. *Neurology* **66**, 1932–1934. 10.1212/01.wnl.0000219759.72195.41 (2006). 16801664 10.1212/01.wnl.0000219759.72195.41

[15] McGarry, J. D., Leatherman, G. F. & Foster, D. W. Carnitine palmitoyltransferase I. The site of inhibition of hepatic fatty acid oxidation by malonyl-CoA. *J. Biol. Chem.* **253**, 4128–4136 (1978). 659409

[16] Rocha, M. C. et al. A novel immunofluorescent assay to investigate oxidative phosphorylation deficiency in mitochondrial myopathy: Understanding mechanisms and improving diagnosis. *Sci. Rep.* **5**, 15037. 10.1038/srep15037 (2015). 26469001 10.1038/srep15037 PMC4606788

[17] Stefanetti, R. et al. Measuring the effects of exercise in neuromuscular disorders: a systematic review and meta-analyses [version 1; peer review: 2 approved]. *Wellcome Open Res.*10.12688/wellcomeopenres.15825.1 (2020). 32671231 10.12688/wellcomeopenres.15825.1 PMC7331112

[18] Zanini, A. et al. Minimum clinically important difference in 30-s sit-to-stand test after pulmonary rehabilitation in subjects with COPD. *Respir. Care* **64**, 1261–1269. 10.4187/respcare.06694 (2019). 31270178 10.4187/respcare.06694

[19] Samsa, G. et al. Determining clinically important differences in health status measures. *Pharmacoeconomics* **15**, 141–155. 10.2165/00019053-199915020-00003 (1999). 10351188 10.2165/00019053-199915020-00003

[20] Jayadevappa, R., Cook, R. & Chhatre, S. Minimal important difference to infer changes in health-related quality of life-a systematic review. *J. Clin. Epidemiol.* **89**, 188–198. 10.1016/j.jclinepi.2017.06.009 (2017). 28676426 10.1016/j.jclinepi.2017.06.009

[21] Rooney, S., McFadyen, D. A., Wood, D. L., Moffat, D. F. & Paul, P. L. Minimally important difference of the fatigue severity scale and modified fatigue impact scale in people with multiple sclerosis. *Mult. Scler. Relat. Disord.* **35**, 158–163. 10.1016/j.msard.2019.07.028 (2019). 31400557 10.1016/j.msard.2019.07.028

[22] Mease, P. J. et al. Estimation of minimum clinically important difference for pain in fibromyalgia. *Arthritis Care Res.* **63**, 821–826. 10.1002/acr.20449 (2011). 10.1002/acr.20449 21312349

[23] Callegari, G. A. et al. Creatine kinase and lactate dehydrogenase responses after different resistance and aerobic exercise protocols. *J. Hum. Kinet.* **58**, 65–72. 10.1515/hukin-2017-0071 (2017). 28828078 10.1515/hukin-2017-0071 PMC5548155

[24] Karaa, A. et al. Randomized dose-escalation trial of elamipretide in adults with primary mitochondrial myopathy. *Neurology* **90**, e1212–e1221. 10.1212/wnl.0000000000005255 (2018). 29500292 10.1212/WNL.0000000000005255 PMC5890606

[25] Madsen, K. L. et al. Safety and efficacy of omaveloxolone in patients with mitochondrial myopathy. *Neurology* **94**, e687. 10.1212/WNL.0000000000008861 (2020). 31896620 10.1212/WNL.0000000000008861 PMC7176297

[26] Frost, A. E. et al. The 6-min walk test (6MW) as an efficacy endpoint in pulmonary arterial hypertension clinical trials: Demonstration of a ceiling effect. *Vascul. Pharmacol.* **43**, 36–39. 10.1016/j.vph.2005.03.003 (2005). 15890561 10.1016/j.vph.2005.03.003

[27] Mota, A. C. et al. Lysosome-dependent LXR and PPARδ activation upon efferocytosis in human macrophages. *Front. Immunol.* **12**, 637778. 10.3389/fimmu.2021.637778 (2021). 34025647 10.3389/fimmu.2021.637778 PMC8137840

[28] Krämer, D. K. et al. Role of AMP kinase and PPARdelta in the regulation of lipid and glucose metabolism in human skeletal muscle. *J. Biol. Chem.* **282**, 19313–19320. 10.1074/jbc.M702329200 (2007). 17500064 10.1074/jbc.M702329200

[29] Longo, N., Frigeni, M. & Pasquali, M. Carnitine transport and fatty acid oxidation. *Biochim. Biophys. Acta* **1863**, 2422–2435. 10.1016/j.bbamcr.2016.01.023 (2016). 26828774 10.1016/j.bbamcr.2016.01.023 PMC4967041

[30] Sebastián, D. et al. Novel role of FATP1 in mitochondrial fatty acid oxidation in skeletal muscle cells. *J. Lipid Res.* **50**, 1789–1799. 10.1194/jlr.M800535-JLR200 (2009). 19429947 10.1194/jlr.M800535-JLR200 PMC2724792

[31] Scimè, A. et al. Rb and p107 regulate preadipocyte differentiation into white versus brown fat through repression of PGC-1α. *Cell Metab.* **2**, 283–295. 10.1016/j.cmet.2005.10.002 (2005). 16271529 10.1016/j.cmet.2005.10.002

[32] Jong-Yeon, K., Hickner, R. C., Dohm, G. L. & Houmard, J. A. Long- and medium-chain fatty acid oxidation is increased in exercise-trained human skeletal muscle. *Metabolism* **51**, 460–464. 10.1053/meta.2002.31326 (2002). 11912554 10.1053/meta.2002.31326

[33] Lai, C. Q. et al. Carbohydrate and fat intake associated with risk of metabolic diseases through epigenetics of CPT1A. *Am. J. Clin. Nutr.* **112**, 1200–1211. 10.1093/ajcn/nqaa233 (2020). 32930325 10.1093/ajcn/nqaa233 PMC7657341

[34] Brejchova, J., Brejchova, K. & Kuda, O. Metabolic pathways of acylcarnitine synthesis. *Physiol. Res.* **73**, 153–163. 10.33549/physiolres.935261 (2024). 10.33549/physiolres.935261 PMC11412349 38752770

[35] Gorman, G. S. et al. Perceived fatigue is highly prevalent and debilitating in patients with mitochondrial disease. *Neuromuscul. Disord.* **25**, 563–566. 10.1016/j.nmd.2015.03.001 (2015). 26031904 10.1016/j.nmd.2015.03.001 PMC4502433

[36] Parikh, S. et al. Fatigue in primary genetic mitochondrial disease: No rest for the weary. *Neuromuscul. Disord.* **29**, 895–902. 10.1016/j.nmd.2019.09.012 (2019). 31653361 10.1016/j.nmd.2019.09.012

[37] Thomas, R. H. et al. Research priorities for mitochondrial disorders: Current landscape and patient and professional views. *J. Inherit. Metab. Dis.* **45**, 796–803. 10.1002/jimd.12521 (2022). 35543492 10.1002/jimd.12521 PMC9429991

[38] Shaibani, A., Frisaldi, E. & Benedetti, F. Placebo response in pain, fatigue, and performance: Possible implications for neuromuscular disorders. *Muscle Nerve* **56**, 358–367. 10.1002/mus.25635 (2017). 28249354 10.1002/mus.25635

[39] Frisaldi, E., Shaibani, A., Benedetti, F. & Pagnini, F. Placebo and nocebo effects and mechanisms associated with pharmacological interventions: An umbrella review. *BMJ Open* **13**, e077243. 10.1136/bmjopen-2023-077243 (2023). 37848293 10.1136/bmjopen-2023-077243 PMC10582987

[40] Kos, D., Duportail, M., D’Hooghe, M., Nagels, G. & Kerckhofs, E. Multidisciplinary fatigue management programme in multiple sclerosis: A randomized clinical trial. *Mult. Scler.* **13**, 996–1003. 10.1177/1352458507078392 (2007). 17623738 10.1177/1352458507078392

[41] Filosto, M. et al. Muscle pain in mitochondrial diseases: A picture from the Italian network. *J. Neurol.* **266**, 953–959. 10.1007/s00415-019-09219-x (2019). 30710167 10.1007/s00415-019-09219-x

[42] Farrar, J. T., Berlin, J. A. & Strom, B. L. Clinically important changes in acute pain outcome measures: A validation study. *J. Pain Symptom Manage.* **25**, 406–411. 10.1016/s0885-3924(03)00162-3 (2003). 12727037 10.1016/s0885-3924(03)00162-3

[43] Kemp, G. J., Taylor, D. J. & Radda, G. K. Control of phosphocreatine resynthesis during recovery from exercise in human skeletal muscle. *NMR. Biomed.* **6**, 66–72. 10.1002/nbm.1940060111 (1993). 8457428 10.1002/nbm.1940060111

[44] Pitceathly, R. D. S., Keshavan, N., Rahman, J. & Rahman, S. Moving towards clinical trials for mitochondrial diseases. *J. Inherit. Metab. Dis.* **44**, 22–41. 10.1002/jimd.12281 (2021). 32618366 10.1002/jimd.12281 PMC8432143

[45] Kidwell, K. M. et al. Application of Bayesian methods to accelerate rare disease drug development: Scopes and hurdles. *Orphanet J. Rare Dis.* **17**, 186. 10.1186/s13023-022-02342-5 (2022). 35526036 10.1186/s13023-022-02342-5 PMC9077995

[46] Mancuso, M., McFarland, R., Klopstock, T., Hirano, M., consortium on Trial Readiness in Mitochondrial, M. International workshop: Outcome measures and clinical trial readiness in primary mitochondrial myopathies in children and adults consensus recommendations. *Neuromuscul. Disord.* **27**, 1126–1137 (2017). 29074296 10.1016/j.nmd.2017.08.006 PMC6094160

[47] Bernstein, M. L. et al. Reanalysis of the 12-minute walk in patients with chronic obstructive pulmonary disease. *Chest* **105**, 163–167. 10.1378/chest.105.1.163 (1994). 8275726 10.1378/chest.105.1.163

[48] Stefanetti, R. et al. Clinical trial dataset supporting: Mavodelpar in Patients withPrimary Mitochondrial Myopathy: A Phase 1 Trial. *Newctle. Univ. Dataset*. (2026). 10.25405/data.ncl.20747032.v1

