# Supplementary material for: Mavodelpar in patients with primary mitochondrial myopathy: a phase 1 trial
Source: Sci Rep. 2026 May 18;16:24058. doi: 10.1038/s41598-026-43287-0 (PMC13439553; doi:10.1038/s41598-026-43287-0)
Supplement: Supplementary file 1 [file 41598_2026_43287_MOESM1_ESM.pdf]

Supplementary File –

Mavodelpar in Patients with Primary Mitochondrial Myopathy: A Phase 1 Trial.
This file contains the Supplementary Results and a summary of the Clinical Trial Protocol.

Table of Contents

Suplementary Results 3

Table S1. Demographics and Baseline Disease Characteristics 4

Table S2. Treatment-Emergent Adverse Events (TEAEs) classified as Gastrointestinal (GI) Disorders (System Organ Class) for the Full Analysis Set – Part A 6

Table S3. Adverse Events Leading to Interruption in Dosing or Discontinuation of Mavodelpar (REN001) During Parts A or B 7

Table S4. Vital signs and ECGs 8

Table S5. Laboratory Parameters 9

Table S6. Subjects with Biochemistry Values of Potential Clinical Importance (PCI) 11

Table S7. Pharmacodynamic (PD) Endpoints 12

Table S8. Muscle Biopsy – Summary Analyses 13

Table S9. Muscle Biopsy – Individual Subject Analyses 14

Muscle Biopsy – Individual Subject Results 15

Muscle Biopsy –Transcriptomic Analysis 16

Table S10. RNA-seq sample metadata and pairing information 14

Figure S1. Transcriptomic Analysis 16

Table S11. Differentially Expressed Genes 17

Table S12. Gene Set Enrichment Analysis 17

Table S13. Modified Fatigue Impact 18

Table S14. Brief Pain Inventory (BPI) – (Short Form) 19

Supplementary References (Results) 20

Study Protocol Summary 21

1.1 Overall Study Design and Study Plan. 22

Figure S2. Study design and assessment schedule. 22

1.1.1 Inclusion and Exclusion Criteria 30

1.1.2 Trial Endpoints and Outcome Measures 230

Table S15. Trial Outcome Measurement Schedule 27

Table S16. Prohibited Medications 29

Table S17. Safety Laboratory Parameters 29

1.1.3 Treatment Discontinuation (Individual Stopping Rules) 30

1.1.4 Replacement of Subjects 300

1.1.5 Dose De-escalation 30

1.1.6 Withdrawal due to Laboratory Test Abnormalities 31

1.1.7 Temporary Discontinuations 32

1.1.8 Review of Safety Data 32

1.1.9 Adjustment of Dose 32

1.2 Statistical Methods 32
1.2.1 Sample Size..... 32
1.2.2 Statistical Analysis Plan..... 32
1.2.3 Disposition of Subjects 32
1.2.4 Analysis Populations..... 33
1.2.5 Safety Analysis 33
1.2.6 Pharmacokinetic Endpoints 33
Supplementary References (Study Protocol Summary) 34

Supplementary Results

Table S1. Demographics and Baseline Disease Characteristics

		Full Analysis Set (N = 23)	Completers Analysis Set (N = 17)[a]
Age [years], mean ± SD (min — max)		54·5 ± 7·8 (43 — 69)	55·2 ± 7·5 (43 — 67)
Men / Women, No. (%)		8 (34·8)/15 (65·2)	5 (29·4)/12 (70·6)
Height [cm], mean ± SD (min — max)		168 ± 12 (151 — 193)	167 ± 11 (151 — 185)
Weight [kg], mean ± SD (min — max)		67·8 ± 16·2 (48·7 — 105·4)	67·8 ± 14·7 (50·2 — 98·8)
BMI [kg/m^2], mean ± SD (min — max)		23·7 ± 4·0 (16·6 — 30·6)	24·3 ± 4·0 (16·6 — 30·6)
**Genetics, No. (%)**	**Heteroplasmy [%],** (min — max)		
m.3243A>G variant[b,c]	7 — 34% blood, 40 — 82% urine, 67 — 85% muscle	11 (47·8)	7 (41·2)
Other pathogenic mtDNA variants		12 (52·2)	10 (58·8)
m.8344A>G	53 — 67% blood, 67% urine	2 (8·7)	2 (11·8)
m.14709T>C	75 — 78% blood, 69% muscle	2 (8·7)	2 (11·8)
m.3761C>A	12% blood, 46% urine, 76% muscle	1 (4·3)	1 (5·9)
m.15699G>C	13% blood, 16% urine, 80% muscle	1 (4·3)	1 (5·9)
m.3250T>C	98% blood, 98% urine, 97% muscle	1 (4·3)	1 (5·9)
m.5690A>G	0% blood, 0% urine, 45% muscle	1 (4·3)	1 (5·9)
m.5703G>A	30% blood, 44% urine, 56% muscle	1 (4·3)	1 (5·9)
m.12271T>C	15% muscle	1 (4·3)	1 (5·9)
m.3256C>T	11% blood, 62% urine	1 (4·3)	0
Single, large-scale mtDNA deletion		1 (4·3)	0
**Race, No. (%)**			
Middle-Eastern		1 (4·3)	1 (5·9)
White		22 (95·7)	16 (94·1)
**Smoking history, No. (%)[d]**			
Never smoked		16 (69·6)	11 (64·7)
Former smoker		4 (17·4)	3 (17·6)
Current smoker		2 (8·7)	2 (11·8)
**Disease characteristics [NMDAS[e]], No. (%)**			
	0 or 1	0	0
	2	10 (43·5)	5 (29·4)
**Myopathy**	3	12 (52·2)	11 (64·7)
	4	1 (4·3)	1 (5·9)
	5	0	0
	0	2 (8·7)	0
	1	11 (47·8)	7 (41·2)
**Exercise intolerance**	2	4 (17·4)	4 (23·5)
	3	5 (21·7)	5 (29·4)
	4	1 (4·3)	1 (5·9)
	5	0	0
	0	2 (8·7)	1 (5·9)
	1	4 (17·4)	2 (11·8)
**Gait stability**	2	11 (47·8)	9 (52·9)
	3	6 (26·1)	5 (29·4)
	4 or 5	0	0
	0	7 (30·4)	4 (23·5)
**Neuropathy**	1	15 (65·2)	12 (70·6)
	2	1 (4·3)	1 (5·9)
	3, 4, or 5	0	0
	0	10 (43·5)	7 (41·2)
	1	3 (13·0)	1 (5·9)
**GI disturbance**	2	4 (17·4)	4 (23·5)
	3	6 (26·1)	5 (29·4)
	4 or 5	0	0
	0	12 (52·2)	7 (41·2)
	1	7 (30·4)	7 (41·2)
**Migraine headaches**	2	3 (13·0)	2 (11·8)
	3 or 4	0	0
	5	1 (4·3)	1 (5·9)

**Diabetes mellitus**	0	12 (52·2)	10 (58·8)
	1	0	0
	2	1 (4·3)	0
	3	1 (4·3)	1 (5·9)
	4	5 (21·7)	5 (29·4)
	5	4 (17·4)	1 (5·9)
**CPEO**	0	13 (56·5)	10 (58·8)
	1	6 (26·1)	5 (29·4)
	2	2 (8·7)	0
	3	1 (4·3)	1 (5·9)
	4	1 (4·3)	1 (5·9)
	5	0	0
**Respiratory muscle weakness**	0	13 (56·5)	9 (52.9)
	1	5 (21·7)	5 (29·4)
	2	1 (4·3)	0
	3	1 (4·3)	1 (5·9)
	4	2 (8·7)	1 (5·9)
	5	1 (4·3)	1 (5·9)
**Cardiovascular system**	0	14 (60·9)	10 (58·8)
	1	4 (17·4)	3 (17·6)
	2	2 (8·7)	2 (11·8)
	3	3 (13·0)	2 (11·8)
	4 or 5	0	0

Abbreviations: BMI, Body Mass Index; CPEO, Chronic Progressive External Ophthalmoplegia; GI, Gastro-intestinal; NMDAS, Newcastle Mitochondrial Disease Adult Scale.

N.B. After database lock, the NMDAS Myopathy score was corrected from 1 to 2 for one subject.

a. Completers Analysis Set: those subjects who completed Part A; the 12 week treatment phase.
b. GenBank RefSeq: NC_012920.1 for human mtDNA.
c. Heteroplasmy is unavailable for n = 2, m.3243A>G subjects.
d. Not available for one participant in both the Full Analysis Set and Completers Analysis Set.
e. NMDAS sub-scales (0-5). Score ranges from 0 (None/Normal) to 5, indicating greater disease seventy. Full description of the NMDAS scale can be found in Schaefer et al. 2006.

Table S2. Treatment-Emergent Adverse Events (TEAEs) classified as Gastrointestinal (GI) Disorders (System Organ Class) for the Full Analysis Set – Part A

Event, frequency (No, subjects).	Subject	Severity, pattern	Onset from treatment, duration	Relatedness to treatment
Constipation (N = 4)	n = 1 (GI history) [a]	Mild, intermittent	Day 54 – 70 (17 days)	Possibly related
	n = 1 (GI history) [b]	Mild, continuous	Day 2 – 8 (6 days)	Possibly related
	n = 1 (GI history) [d]	Mild, intermittent	Day 10 – 16 (7 days)	Not-related
	n = 1 [h]	Mild, continuous	Ongoing	Possibly related
Gastroesophageal reflux (N = 3)	N = 1 (GI history) [c]	Mild, intermittent	Day 2, ongoing	Possibly related
	n = 1 [e]	Mild, intermittent	Day 3, ongoing	Probably related
	n = 1 (GI history) [a]	Mild, intermittent	Day 54 – 70 (17 days)	Possibly related
Diarrhoea (N = 2)	n = 1 [f]	Moderate, intermittent	Day 10 – 21 (12 days)	Possibly related
	n = 1 [g]	Moderate, intermittent	Day 54 (<1 day)	Possibly related
Nausea (N = 2)	n = 1 (GI history) [c]	Mild, intermittent	Day 2, ongoing	Possibly related
	n = 1 [g]	Mild, intermittent	Day 2 – 5 (4 days)	Possibly related
Dyspepsia (N = 1)	n = 1 (GI history) [a]	Moderate, intermittent	Day 53 – 71 (19 days)	Possibly related
Lip ulceration (N = 1)	n = 1 (GI history) [a]	Mild, continuous	Day 39– 45 (7 days)	Not related: Viral illness
Mouth ulceration (N = 1)	n = 1 (GI history) [a]	Mild, continuous	Day 49 – 55 (7 days)	Not related: Viral illness
Salivary hypersecretion (N = 1)	n = 1 (GI history) [b]	Mild, intermittent	Ongoing	Possibly related
Abdominal pain (N = 1)	n = 1 (GI history) [b]	Moderate, intermittent	Ongoing	Possibly related
Frequent bowel movements (N = 1)	n = 1 [e]	Mild, intermittent	Day 3, ongoing	Probably related
Lip dry (N = 1)	n = 1 [f]	Mild, continuous	Ongoing	Possibly related
Abdominal pain upper (N = 1)	n = 1 [i]	Mild, intermittent	Day 8 – 15 (8 days)	Not related: ran out of Rifaximin tablets
Total = 19 TEAEs	N = 9 patients, 39.1%	Mild (N = 15/19), 78.9%<br>Moderate (N = 4/19), 21.1%<br>Intermittent (N = 14/19), 73.7%<br>Moderate (N = 5/19), 26.3%		Possible (N =13/19), 68.4%<br>Probably (N =2/19), 10.5%<br>Not-related (N =4/19), 21.1%

Treatment-Emergent Adverse Events (TEAEs) classified as Gastrointestinal (GI) Disorders (System Organ Class) for the Full Analysis Set (N = 23). A total of 19 TEAEs were reported in 9 subjects (39·1%). Of the 19 TEAEs, 15 (79%) were classified as mild, and 14 (74%) were intermittent in occurrence. Four of the 19 TEAEs (21.1%) were not related to treatment; 13 (68.4%) were deemed possible-related, and 2 (10.5%) were considered probably-related. Six of the nine subjects reported >1 GI Disorders TEAE (a, b, c, e, f, g), three of whom had a prior GI medical history (a, b, c). Three subjects reported a single GI Disorder TEAE (d, h, i), one of whom had a prior GI medical history (d).

a. n = 5 GI Disorders TEAEs experienced (constipation, gastroesophageal reflux, dyspepsia, lip ulceration, mouth ulceration). Subject had an ongoing GI medical history of dysphagia and Gastroesophageal reflux.
b. n =3 GI Disorders TEAEs experienced (constipation, salivary hypersecretion, abdominal pain). Subject had an ongoing GI medical history of irritable bowel syndrome.
c. n = 2 GI Disorders TEAEs experienced (gastroesophageal reflux, nausea). Subject had an ongoing GI medical history of gastrointestinal motility disorder.
d. n = 1 GI Disorders TEAE experienced (constipation). Subject had an ongoing GI medical history of constipation.
e. n = 2 GI Disorders TEAEs experienced (gastroesophageal reflux and frequent bowel movements), no GI medical history.
f. n = 2 GI Disorders TEAEs experienced (diarrhoea and lip dry), no GI medical history.
g. n = 2 GI Disorders TEAEs experienced (diarrhoea and nausea), no GI medical history.
h. n = 1 GI Disorders TEAE experienced (constipation), no GI medical history.
i. n = 1 GI Disorders TEAE experienced (abdominal pain upper), no GI medical history.

Table S3. Adverse Events Leading to Interruption in Dosing or Discontinuation of Mavodelpar (REN001) During Parts A or B

Sex, genotype	Adverse Event	Onset Day	Duration	Relatedness to Treatment	Treatment Break	Outcome
Female, m.3243A>G[a]	Pain in extremity (Left leg pain)	Day 59	8 days	Possibly related	6 days	Recovering/resolving
Female, m.12271T>C[b]	Dyspepsia (2 events)	Day 53 & Day 85	19 days; 10 days	Possibly related	6 days	Recovered/resolved
	Tongue ulceration	Day 85	4 days	Possibly related		Recovered/resolved
	IBS	Day 88	Ongoing	Possibly related		Not recovered/resolved
Female, m.3243A>G	ALT increased	Day 11	5 days	Probably related	Discontinued (Day 21)[c]	Recovered/resolved
	AST increased	Day 11	5 days	Probably related		Recovered/resolved
	Blood CK increased	Day 9	7 days	Probably related		Recovered/resolved

Abbreviations: ALT, Alanine aminotransferase; AST, Aspartate aminotransferase; CK, Creatine kinase; IBS, Irritable bowel syndrome.

a. Temporary discontinuation after 59 days of treatment. Following a six day temporary break, the subject restarted mavodelpar (for a further 21 days). However, as their pain continued, the subject decided to not continue into Part B.
b. Temporary discontinuation after 63 days of treatment. Following a six day temporary break, the subject restarted mavodelpar (for a further 19 days). However, subject was withdrawn in Part B upon recurrence of gastrointestinal symptoms (physicians' decision).
c. Discontinued (without re-starting) after 10 days of treatment due to logistical reasons (being unable to commit to daily blood tests required for monitoring following an increase in CK).

Table S4. Vital signs and ECGs

	Full Analysis Set (N = 23)		Completers Analysis Set (N = 17)[a]				
		**Baseline**		**Baseline**	**Week 12**	**Change from Baseline**	***P* value**
	n	Mean ± SD	n	Mean ± SD	Mean ± SD	Mean ± SD (95% CI)	
**Vital Signs**[b]							
SBP (mmHg)[c]	23	124 ± 13	17	126 ± 14	123 ± 11	-3 ± 10 (-7 to 2)	0·40
DBP (mmHg)[c]	23	75·4 ± 7·1	17	76·1 ± 7·8	72·8 ± 8·9	-3·2 ± 5·8 (-6·2 to -0·3)	0·12
Pulse rate (bpm)[d]	23	72·8 ± 13·2	17	75·8 ± 13·8	77·9 ± 12·9	2·1 ± 10·3 (-3·3 to 7·4)	0·42
Body temperature (°C)	23	36·6 ± 0·3	17	36·6 ± 0·3	36·7 ± 0·3	0·1 ± 0·3 (0·0 to 0·3)	0·12
Body weight (kg)	23	67·7 ± 16·6	17	67·4 ± 15·2	68·7 ± 14·7	1·4 ± 1·8 (-0·5 to 2·3)	0·30
**12-lead ECG**[e]							
HR (bpm)	23	70·9 ± 11·9	17	72·6 ± 11·8	74·8 ± 12·7	2·2 ± 6·4 (-1·1 to 5·5)	0·49
Females	15	70·6 ± 12·4	12	71·3 ± 12·2	74·8 ± 12·8	3·5 ± 6·6 (-0·7 to 7·7)	0·40
Males	8	71·5 ± 11·8	5	75·6 ± 11·7	74·6 ± 14·0	-1·0 ± 5·1 (-7·4 to 5·4)	0·81
RR Interval (msec)	23	869 ± 158	17	846 ± 150	826 ± 159	-20 ± 74 (-58 to 18)	0·49
Females	15	874 ± 169	12	862 ± 158	826 ± 165	-36 ± 74 (-83 to 11)	0·40
Males	8	860 ± 145	5	810 ± 135	828 ± 161	18 ± 66 (-64 to 101)	0·80
PR Interval (msec)	23	148 ± 20	17	149 ± 20	150 ±21	0 ± 7 (-3 to 4)	0·81
Females	15	145 ± 21	12	146 ± 21	148 ± 22	2 ± 8 (-4 to 7)	0·71
Males	8	153 ± 17	5	156 ± 17	154 ± 21	-2 ± 4 (-7 to 2)	0·80
QRS Interval (msec)	23	91·9 ± 8·7	17	91·7 ± 7·9	86·3 ± 9·9	-5·4 ± 9·6 (-10·3 to -0·4)	0·28
Females	15	87·9 ± 6·7	12	89·1 ± 6·2	84·8 ± 9·9	-4·3 ± 10·3 (-10·9 to 2·2)	0·40
Males	8	99·3 ± 7·3	5	97·8 ± 9·0	90·0 ± 10·0	-7·8 ± 8·4 (-18·2 to 2·6)	0·77
QT Interval: (msec)	23	396 ± 30	17	390 ± 27	388 ± 30	-3 ± 16 (-11 to 6)	0·71
Females	15	394 ± 24	12	397 ± 25	393 ± 30	-3 ± 18 (-15 to 8)	0·71
Males	8	399 ± 40	5	375 ± 27	374 ± 26	-1 ± 10 (-13 to 11)	0·86
QTcF Interval (msec)	23	415 ± 16	17	413 ± 16	414 ± 17	1 ± 13 (-6 to 7)	0·81
Females	15	416 ± 15	12	418 ± 15	420 ± 14	2 ± 15 (-7 to 12)	0·71
Males	8	414 ± 19	5	403 ± 13·	400 ± 17	-3 ± 7 (-12 to 6)	0·80

Abbreviations: DBP, Diastolic blood pressure; ECG, electrocardiogram; HR, heart rate; QTcF, heart rate corrected QT interval using the Fredericia formula; SBP, Systolic blood pressure.

N.B: Post-hoc analyses (after database lock), not specified in the statistical analysis plan, have been included for male and female ECG subgroups.

a. Completers Analysis Set: those subjects who completed Part A; the 12 week treatment phase. Only those subjects with data at both Baseline and Week 12 are included in summaries.
b. Measured prior to dosing, after at least 5 minutes of supine rest.
c. Measured in the dominant arm.
d. Measured in the brachial/radial artery (dominant arm).
e. Measured prior to dosing, after at least 10 minutes of supine rest.

Table S5. Laboratory Parameters

	Normal range		Full Analysis Set (N = 23)		Completers Analysis Set (N = 17)[a]			
			Baseline		Baseline	Week 12	Change from Baseline	*P* value
		n	Mean ± SD	n		Mean ± SD	Mean ± SD (95% CI)	
**Biochemistry**								
Sodium (mmol/L)	135 - 145	23	140 ± 2	17	141 ± 2	139 ± 2	-2 ± 1 (-3 to -1)	**0·004**
Potassium (mmol/L)	3·5 - 5·1	23	4·38 ± 0·39	15	4·40 ± 0·39	4·55 ± 0·44	0·15 ± 0·31 (-0·02 to 0·32)	0·38 *
Calcium (mmol/L)	2·2 - 2·6	23	2·34 ± 0·10	17	2·35 ± 0·09	2·35 ± 0·11	0·00 ± 0·07 (-0·04 to 0·04)	1·0 *
Urea (mmol/L)	1·7 - 8·3	23	6·05 ± 1·41	17	6·29 ± 1·54	6·34 ± 1·29	0·04 ± 1·36 (-0·66 to 0·74)	0·99 *
Uric acid (µmol/L)		23	303 ± 60	17	300 ± 57	314 ± 84	14 ± 42 (-8 to 36)	0·47
Female	175 - 363	15	286 ± 44	12	289 ± 44	293 ± 69	4 ± 38 (-20 to 28)	0·78
Male	266 - 474	8	336 ± 76	5	328 ± 80	366 ± 100	38 ± 46 (-19 to 95)	0·45
CRP (mg/L)	0 - 5	23	3·50 ± 4·90	17	3·24 ± 3·74	2·59 ± 2·37	-0·64 ± 1·94 (-1·64 to 0·36)	0·47 *
Creatinine (µmol/L)		23	66·1 ± 14·2	17	64·6 ± 13·3	61·9 ± 15·3	-2·7 ± 8·3 (-7·0 to 1·5)	0·47
Female	49 - 92	15	63·1 ± 10·9	12	62·9 ± 11·9	58·2 ± 14·0	-4·8 ± 8·7 (-10·2 to 0·7)	0·40
Male	66 - 112	8	71·8 ± 18·4	5	68·6 ± 16·9	70·8 ± 16·2	2·2 ± 5.0 (-4.0 to 8·4)	0·53
Creatine kinase (IU/L)		23	229 ± 236	16	191 ± 139	216 ± 176	25 ± 160 (-61 to 110)	0·63
Female	26 - 140	15	170 ± 123	11	158 ± 108	200 ± 182	42 ± 178 (-78 to 162)	0·56
Male	38 - 204	8	339 ± 352	5	262 ± 185	249 ± 177	-12 ± 120 (-161 to 136)	0·83
Glucose[b] (mmol/L)	3·5 - 7·9	23	7·91 ± 3·69	17	7·76 ± 3·62	8·79 ± 5·18	1·03 ± 2·36 (-0·19 to 2·24)	0·46 *
Triglycerides (mmol/L)	0 - 2·2	23	1·77 ± 0·84	17	1·98 ± 0·86	2·34 ± 1·91	0·36 ± 1·33 (-0·33 to 1·04)	0·53 *
Troponin I (ng/L)								
Female	0 - 15·6	15	All levels < 10ng/L	17	All levels < 10ng/L	< 10ng/L (n = 16); 14 (n = 1)	NA	NA
Male	0 - 34·2							
Total Bilirubin (µmol/L)	0 - 20	23	6·13 ± 3·83	17	5·06 ± 2·95	4·53 ± 2·15	-0·53 ± 1·91 (-1·51 to 0·45)	0·61 *
Total protein (g/L)	63 - 83	23	68·1 ± 4·0	17	68·9 ± 3·7	68·8 ± 4·4	-0·1 ± 3·4 (-1·9 to 1·6)	0·99 *
Lipase (U/L)	13 - 60	23	41·7 ± 19·8	17	41·9 ± 20·6	44·6 ± 21·3	2·7 ± 24·1 (-9·7 to 15·1)	0·87 *
Albumin (g/L)	34 - 50	23	44·3 ± 2·8	17	44·3 ± 3·0	43·9 ± 3·0	-0·4 ± 2·3 (-1·5 to 0·8)	0·47 *
Aldolase (IU/L)	0 - 7·6	23	4·03 ±1·67	17	3·81 ± 1·40	3·99 ± 1·27	0·19 ± 1·71 (-0·69 to 1·07)	0·61 *
ALP (IU/L)		23	73·2 ± 21·2	17	77·0 ± 21·8	65·3 ± 17·5	-11·7 ± 13·2 (-18·5 to -4·9)	**0·02**
Female	35 - 104	15	78·1 ± 23·0	12	79·7 ± 24·3	68·8 ± 19·0	-10·9 ± 15·59 (-20·8 to -1·0)	0·27
Male	40 - 129	8	64·1 ± 14·5	5	70·6 ± 14·1	57·0 ± 11·0	-13·6 ± 5·1 (-19·9 to -7·3)	0·08
AST (IU/L)		22	26·0 ± 12·3	13	24·5 ± 8·0	27·6 ± 11·2	3·2 ± 13·7 (-5·2 to 11·5)	0·63
Female	0 - 31	15	22·9 ± 6·4	8	22·4 ± 4·9	29·8 ± 13·8	7·4 ± 13·3 (-3·8 to 18·5)	0·46
Male	0 - 37	8	31·8 ± 18·2	5	27·8 ± 11·4	24·2 ± 4·5	-3·6 ± 12·8 (-19·5 to 12·3)	0·63
ALT (IU/L)		23	25·5 ± 11·7	17	24·2 ± 10·1	25·4 ± 12·9	1·2 ± 13·4 (-5·7 to 8·1)	0·93
Female	10 - 35	15	20·1 ± 5·4	12	19·7 ± 4·1	23·8 ± 13·3	4·2 ± 14·2 (-4·83 to 13·2)	0·56
Male	10 - 50	8	35·5 ± 13·9	5	35·0 ± 12·43	29·2 ± 12·4	-5·8 ± 8·9 (-16·9 to 5·3)	0·45
GGT (IU/L)		23	16·9 ± 8·2	17	17·8 ± 8·9	15·1 ± 8·7	-2·7 ± 3·4 (-4·5 to -0·9)	**0·04**
Female	6 - 42	15	15·9 ± 7·7	12	16·3 ± 8·4	13·8 ± 6·9	-2·5 ± 3·7 (-4·8 to -0·2)	0·27
Male	10 - 71	8	18·6 ± 9·2	5	21·4 ± 9·9	18·2 ± 12·4	-3·2 ± 3·1 (-7·1 to 0·7)	0·40
Total cholesterol (mmol/L)	2·3 - 4·9	23	4·77 ± 0·87	17	4·95 ± 0·84	5·12 ± 0·80	0·18 ± 0·58 (-0·12 to 0·48)	0·48 *
**Haematology**								
MCV platelet count (fL)	80 – 99	23	88·0 ± 3·9	16	87·6 ± 4·3	88·2 ± 4·9	0·5 ± 1·8 (-0·4 to 1·5)	0·58
Red cell count (10^12/L)		23	4·45 ± 0·43	16	4·47 ± 0·43	4·31 ± 0·30	-0·16 ± 0·25 (-0·30 to -0·03)	0·13
Female	3·95 - 5·15	15	4·33 ± 0·41	11	4·36 ± 0·36	4·20 ± 0·24	-0·16 ± -0·28 -0·35 to 0·03)	0·18
Male	4·4 - 5·8	8	4·68 ± 0·39	5	4·72 ± 0·50	4·55 ± 0·32	-0·17 ± 0·2 -0·42 to 0·08)	0·39
Reticulocytes (%)		23	1·30 ± 0·23	16	1·32 ± 0·26	1·41 ± 0·34	0·10 ± 0·19 (-0·00 to 0·20)	0·18
Female	0·45 - 2·42	15	1·30 ± 0·17	11	1·31 ± 0·18	1·48 ± 0·29	0·17 ± 0·16 0·07 to 0·28)	**0·024**
Male	0·38 - 2·64	8	1·29 ± 0·33	5	1·33 ± 0·42	1·27 ± 0·43	-0·07 ± 0·17 -0·27 to 0·14)	0·68
Haemoglobin (g/L)		23	135 ± 12	16	134 ± 11	129 ± 11	-5·75 ± 7·37 (-9·68 to -1·82)	0·08
Female	115 - 155	15	130 ± 9	11	130 ± 8	125 ± 11	-4·73 ± 8·28 (-10·29 to 0·84)	0·18
Male	130 - 170	8	144 ± 11	5	144 ± 13	136 ± 9	-8 ± 4·8 (-13·95 to -2·05)	0·24
Haematocrit (%)		23	39·1 ± 3·4	16	39·1 ± 3·4	37·9 ± 3·0	-1·2 ± 2·2 (-2·3 to 0·0)	0·18
Female	33 - 45	15	37·9 ± 2·7	11	38·0 ± 2·1	37·2 ± 2·8	-0·9 ± 2·3 (-2·4 to 0·7)	0·41
Male	37 - 50	8	41·2 ± 3·7	5	41·4 ± 4·7	39·6 ± 3·0	-1·8 ± 1·8 (-4·0 to 0·4)	0·32
Platelets (10^9/L)	150 - 400	23	248 ± 50	16	243 ± 56	248 ± 75	6 ± 38 (14 to 26)	1·00 *
Leukocytes (10^9/L)	3 - 10	23	6·22 ± 1·33	16	6·00 ± 1·26	5·79 ± 1·22	-0·21 ± 1·48 (-1·00 to 0·58)	0·58 *
Neutrophils (10^9/L)	2 - 7·5	23	3·77 ± 1·18	16	3·65 ± 1·17	3·36 ± 1·07	-0·29 ± 1·29 (-0·98 to 0·39)	0·51 *
Lymphocytes (10^9/L)	1·2 - 3·65	23	1·69 ± 0·43	16	1·63 ± 0·47	1·67 ± 0·52	0·04 ± 0·30 (-0·12 to 0·20)	0·51 *
Monocytes (10^9/L)	0·2 - 1	23	0·54 ± 0·17	16	0·51 ± 0·12	0·54 ± 0·11	0·03 ± 0·07 (-0·01 to 0·07)	0·26 *
Eosinophils (10^9/L)	0 - 0·4	23	0·17 ± 0·09	16	0·16 ± 0·09	0·17 ± 0·10	0·01 ± 0·04 (-0·01 to 0·03)	0·51 *
Basophils (10^9/L)	0 - 0·1	23	0·06 ± 0·02	16	0·05 ± 0·01	0·06 ± 0·02	0·01 ± 0·01 (0·00 to 0·01)	0·15 *
**Urinalysis**								
pH	4·5 - 8·0	23	5·87 ± 0·79	17	5·88 ± 0·76	5·88 ± 0·63	0·00 ± 0·92 (-0·47 to 0·47)	1

Protein Microscopy							
Negative		21 (91·3)		16 (94·1)	14 (82·3)		
Trace	23	2 (8·7)	17	1 (5·9)	2 (11·8)	NA	NA
+ (0·5 g/L)		0		0	1 (5·9)		
++ (0·5 g/L)		0		0	0		
Glucose Microscopy							
Negative		17 (73·0)		13 (76·5)	13 (76·5)		
Trace (2·3 mmol/L)	23	1 (4·3)	17	1 (5·9)	0	NA	NA
+++ (22·3 mmol/L)		1 (4·3)		1 (5·9)	1 (5·9)		
++++ (≥ 55 mmol/L)		4 (17·4)		2 (11·8)	3 (17·6)		
Ketones Microscopy							
Negative	23	23 (100)	17	17 (100)	17 (100)	NA	NA
Blood hCG Microscopy							
Negative		19 (82·6)		14 (82·4)	15 (88·2)		
Trace	23	1 (4·3)	17	1 (5·9)	0	NA	NA
+		2 (8·7)		1 (5·9)	2 (11·8)		
++		1 (4·3)		1 (5·9)	0		

Abbreviations: ALP, Alkaline phosphatase; ALT, Alanine aminotransferase; AST, Aspartate aminotransferase; CRP, C-reactive protein; GGT, Gamma glutamyl transferase; hCG, human chorionic gonadotropin; MVC, Mean corpuscular volume; NA, not applicable.

N.B: Data analysis (after database lock) not specified in the statistical analysis plan included male/female subgroups. * No significant differences in either sex (data not presented).

a. Completers Analysis Set: those subjects who completed Part A; the 12 week treatment phase. Only those subjects with data at both Baseline and Week 12 are included in summaries.
b. Non-fasted.

Table S6. Subjects with Biochemistry Values of Potential Clinical Importance (PCI)

Sex, genotype	Last Dose Day	Onset Day (Analysis Week)	Increase from Baseline	Values
**CK (IU/L)[a]**				
Female, m.3243A>G[c]	Day 10	Day 9 (Week 1)<br>Day 11 (Unscheduled)<br>Day 15 (Week 2)<br>Day 21 (Early Termination)	5·1-fold<br>8.0-fold<br>1·4-fold<br>0.8-fold	Baseline: 198 IU/L, Day 9: 1011 IU/L*<br>Day 11: 1577 IU/L*<br>Day 15: 271 IU/L<br>Day 21: 160 IU/L
Female, m.12271T>C[d]	Day 88	Day 7 (Week 1)<br>Day 14 (Week 2)<br>Day 28 (Week 4)<br>Day 56 (Week 8)<br>Day 66 (Unscheduled)	10·0-fold<br>3·0-fold<br>1·9-fold<br>2·1-fold<br>3·1-fold	Baseline: 95 IU/L, Day 7: 949 IU/L*<br>Day 14: 283 IU/L<br>Day 28: 181 IU/L<br>Day 56: 198 IU/L<br>Day 66: 292 IU/L
**Troponin I (ng/L)[b]**				
Male, m.3243A>G	Day 280	Day 9 (Week 1)<br><10.0 ng/L for all other assessments	9·7-fold	Baseline: <10·0 ng/L, Day 9: 97·4 ng/L
**Aldolase (IU/L)[b]**				
Female, m.3243A>G	Day 14	Day 7 (Week 1)	1·5-fold	Baseline: 5·6 IU/L, Day 7: 8·5 IU/L
Female, m.3243A>G	Day 15	Day 11 (Unscheduled)	1.9-fold	Baseline: 4·4 IU/L, Day 11: 8·4 IU/L
Female, m.15699G>C	Day 85	Day 29 (Week 4)	3.0-fold	Baseline: 2·9 IU/L, Day 29: 8·7 IU/L
Female, m.5690A>G	Day 230	Day 9 (Week 1)	3.0-fold	Baseline: 2·7 IU/L, Day 9: 8·1 IU/L
Male, m.5703G>A	Day 175	Day 11 (Week 1)	1·6-fold	Baseline: 5·0 IU/L, Day 11: 7·8 IU/L
Female, m.12271T>C[b]	Day 88	Day 7 (Week 1)<br>Day 14 (Week 2)	3·1-fold<br>2·3-fold	Baseline: 5·2 IU/L, Day 7: 16·2 IU/L<br>Day 14: 12·0 IU/L
Female, m.14709T>C	Day 154	Day 6 (Week 1)	2·6-fold	Baseline: 3·0 IU/L, Day 6: 7·7 IU/L
Female, m.8344A>G	Day 229	Day 28 (Week 4)	3·3-fold	Baseline: 5·1 IU/L, Day 28: 16·6 IU/L

Data is individual subjects with biochemistry values of Potential Clinical Importance (PCI). Normal range of Creatine kinase (CK): 26 – (140 IU/L) (Females); Troponin I: 0 – 34·2 (ng/L) (Males) and Aldolase 0 – 7·6 (IU/L).

Abbreviations: CK, Creatine kinase.

a. PCI was defined as either (normal at baseline and post-baseline value 5-fold > Upper Limit Normal (ULN)) or (>ULN at baseline and post-baseline 5-fold value 5-fold > baseline). * > 5-fold higher than ULN
b. PCI was defined as >ULN.
c. Subject discontinued (without re-starting) after 10 days of treatment due to logistical reasons (being unable to commit to daily blood tests required for monitoring following an increase in CK.
d. This subject had study drug interruption (for 6 days) starting Day 64, due to dyspepsia. Following this temporary break, the subject restarted mavodelpar (for a further 19 days). However, subject was withdrawn in Part B upon recurrence of gastrointestinal symptoms (physicians' decision).

Table S7. Pharmacodynamic (PD) Endpoints

	Full Analysis Set (N = 23)		Completers Analysis Set[a] (N = 17)				
		Baseline		Baseline	Week 12	Change from Baseline	*P* value
	n	Mean ± SD	n	Mean ± SD	Mean ± SD	Mean ± SD (95% CI)	
**Serum Biomarkers**							
FGF-21 (pg/mL)[b]	18	710 ± 599	16	662 ± 620	651 ± 575	-10 ± 174 (-103 to 82)	0·81
m.3243A>G	8	933 ± 802	6	879 ± 941	922 ± 887	43 ± 181 (-147 to 233)	0·58
Other pathogenic mtDNA variants	10	532 ± 313	10	532 ± 313	489 ± 187	-43 ± 171 (-165 to 80)	0·45
GDF-15 (pg/mL)[c]	18	2026 ± 1212	16	1953 ± 1154	2093 ± 1027	141 ± 287 (-1 to 294)	0·14
m.3243A>G	8	2130 ± 1401	6	1968 ± 1333	2109 ± 1253	141 ± 185 (-53 to 335)	0·12
Other pathogenic mtDNA variants	10	1943 ± 1110	10	1943 ± 1110	2084 ± 941	140 ± 344 (-106 to 387)	0·23
**Acylcarnitine panel** (µmol/L)							
Acetyl-carnitine (C2)	21	21·8 ± 5·1	14	20·9 ± 4·7	23·1 ± 4·5	2·2 ± 6·4 (-1·5 to 5·9)	0·33
Butyryl-carnitine (C4)	21	0·31 ± 0·11	14	0·32 ± 0·13	0·28 ± 0·11	-0·04 ± 0·11 (-0·10 to 0·02)	0·33
Free-carnitine (C0)	21	29·8 ± 9·1	15	29·7 ± 9·0	31·7 ± 8·9	2·0 ± 8·2 (-2·6 to 6·6)	0·36
Hexanoyl-carnitine (C6)	21	0·08 ± 0·02	14	0·07 ± 0·01	0·07 ± 0·02	0·00 ± 0·02 (-0·01 to 0·01)	1·00
Hydroxybutryl-carnitine (C4)	18	0·14 ± 0·05	9	0·13 ± 0·04	0·15 ± 0·05	0·02 ± 0·05 (-0·02 to 0·06]	··
Isovaleryl-carnitine (C5)	21	0·30 ± 0·09	14	0·32 ± 0·10	0·26 ± 0·08	-0·06 ± 0·08 (-0·10 to -0·01)	**0·01**
Octanoyl-carnitine (C8)	21	0·13 ± 0·06	15	0·13 ± 0·08	0·15 ± 0·10	0·02 ± 0·04 (0·00 to 0·04)	**0·046**
Palmitoyl-carnitine (C16)	21	0·95 ± 0·29	14	1·01 ± 0·27	1·04 ± 0·26	0·03 ± 0·30 (-0·14 to 0·20)	0·73
Propionyl-carnitine (C3)	21	1·24 ± 0·40	14	1·25 ± 0·38	1·50 ± 0·46	0·25 ± 0·24 (0·11 to 0·39)	**0·002**
Tetradecenyl-carnitine (C14)	21	0·10 ± 0·04	14	0·10 ± 0·04	0·09 ± 0·05	-0·01 ± 0·06 (-0·05 to 0·03)	0·66

Abbreviations: C, length of the carbon chain in the acylcarnitine molecule; FGF-21, fibroblast growth factor 21; GDF-15, growth/differentiation factor 15.

a. Completers Analysis Set: those subjects who completed Part A; the 12 week treatment phase. Only those subjects with data at both Baseline and Week 12 are included in summaries.
b. Abnormal values defined as >200 pg/m[1]
c. GDF-15 levels previously stratified as normal = <1200 pg/mL[2]

Table S8. Muscle Biopsy – Summary Analyses

	Full Analysis Set (N = 12)		Completers Analysis Set[a] (N = 10)				
		Baseline		Baseline	Week 12	Change from Baseline	*P* value
	n	Mean ± SD	n	Mean ± SD	Mean ± SD	Mean ± SD (95% CI)	
**mtDNA copy number (per µm^2)**	12	56·65 ± 59·03	10	61·46 ± 63·90	59·44 ± 39·14	-2·02 ± 30·05 (-23·52 to 19·47)	0·84
**mtDNA heteroplasmy level (%)[b]**	12	63·64 ± 21·67	10	63·6 ± 22·84	66·7 ± 20·57	3·1 ± 8·82 (-3·21 to 9·41)	0·30
m.3243A>G	2	69·50 ± 3·54	2	69·50 ± 3·54	73·50 ± 2·12	4·00 ± 5·66 (-46·82 to 54·82)	0·5
Other rare mDNA variants	10	62·33 ± 23·98	8	62·13 ± 25·63	65·00 ± 22·95	2·88 ± 9·76 (-5·29 to 11·04)	0·76
**COX-deficient fibres (%)**	12	6·42 ± 10·8	10	6·6 ± 10·82	5·2 ± 7·19	-1·4 ± 10·91 (-9·20 to 6·40)	0·69
**COX-deficient RRF (%)**	12	2·17 ± 4·00	10	2·4 ± 4·35	1·6 ± 2·67	-0·8 ± 4·44 (-3·98 to 2·38)	0·58
**Complex I (CI)**							
CI Positive (%)	12	58·35 ± 35·31	10	53·63 ± 36·63	53·26 ± 32·05	-0·36 ± 18·15 (-13·34 to 12·62)	0·95
CI Intermediate + (%)	12	9·72 ± 9·75	10	10·58 ± 10·47	10·98 ± 10·83	0·40 ± 14·02 (-9·63 to 10·43)	0·93
CI Intermediate - (%)	12	5·80 ± 5·69	10	6·55 ± 5·95	3·99 ± 3·21	-2·56 ± 4·86 (-6·04 to 0·92)	0·13
CI Negative (%)	12	26·10 ± 31·26	10	29·25 ± 33·34	31·77 ± 34·53	2·52 ± 7·02 (-2·50 to 7·54)	0·29
**Complex IV (CIV)**							
CIV Positive (%)	12	93·89 ± 10·56	10	93·07 ± 11·48	90·58 ± 15·41	-2·49 ± 17·63 (-15·10 to 10·13)	0·67
CIV Intermediate + (%)	12	3·54 ± 8·45	10	4·05 ± 9·25	4·23 ± 7·65	0·17 ± 12·41 (-8·71 to 9·05)	0·97
CIV Intermediate - (%)	12	1·26 ± 2·03	10	1·41 ± 2·21	2·47 ± 4·20	1·06 ± 4·38 (-2·07 to 4·19)	0·46
CIV Negative (%)	12	1·31 ± 1·89	10	1·47 ± 2·05	2·72 ± 4·36	1·25 ± 3·48 (-1·24 to 3·74)	0·28
**ATP (nmol/mg)**	12	4·22 ± 1·83	10	4·25 ± 2·02	3·58 ± 1·13	-0·68 ± 1·91 (-2·04 to 0·69)	0·29
**ADP (nmol/mg)**	12	0·77 ± 0·28	10	0·77 ± 0·31	0·67 ± 0·21	-0·11 ± 0·29 (-0·32 to 0·11)	0·29
**ATP/ADP ratio**	12	5·37 ± 1·25	10	5·34 ± 1·38	5·38 ± 0·66	0·04 ± 1·82 (-1·27 to 1·34)	0·95
**CPT1B expression**							0·60
CPT1B above (%)[b]	12	12·41 ± 15·50	10	13·47 ± 16·87	16·33 ± 15·16	2·87 ± 16·66 (-9·05 to 14·78)	0·06
CPT1B below (%)[b]	12	3·68 ± 6·49	10	4·13 ± 7·05	0·84 ± 2·53	-3·29 ± 4·82 (-6·74 to 0·16)	0·45
**Median Porin/fibre[c]**	12	0·79 ± 0·04	10	0·79 ± 0·04	0·77 ± 0·03	-0·02 ± 0·06 (-0·06 to 0·03)	0·84

Summary of muscle biopsy data, including mtDNA copy number, mtDNA heteroplasmy, Complex I & IV protein level classification, ATP/ADP levels, CPT1B protein expression and mitochondrial mass (median porin).
Abbreviations: CPT1B, Carnitine Palmitoyltransferase; CI, Complex I; CIV, Complex IV; RRF, ragged-red fibres.

a. Completers Analysis Set: those subjects who completed Part A; the 12 week treatment phase. Only those subjects with data at both Baseline and Week 12 are included in summaries.
b. CPT1b above and below reflects the percentage of fibres with a higher or lower than expected CPT1b fluorescence, respectively (when compared to control muscle tissue- sampled from non-mitochondrial individuals, for skeletal muscle assay comparison purposes only).
c. Fluorescence intensity per µm

Table S9. Muscle Biopsy – Individual Subject Analyses

Pathogenic mtDNA variant	ID/Timepoint	mtDNA copy No. (μm^2)	mtDNA heteroplasmy (%)	CI Pos (%)	CI Int+ (%)	CI Int- (%)	CI Neg (%)	CIV Pos (%)	CIV Int+ (%)	CIV Int- (%)	CIV Neg (%)	ATP/ADP ratio	CPT1B above (%)	CPT1B below (%)	Median Porin/ Area (fibre)
m.3243A>G	01 – B	47·46	67%	66·24	14·41	5·16	14·19	98·49	0·97	0·32	0·22	6·5	31·56	0	0·791
	01 - 12 W	38·65	75%	71·61	17·42	3·87	7·1	99·35	0	0·65	0	5·07	15·47	0	0·760
m.3761C>A	02 - B	75·46	76%	14·38	10·63	10	65	100	0	0	0	7·25	6·31	2·7	0·789
	02 - 12 W	80·34	80%	7·7	8·21	6·9	77·19	99·94	0	0·06	0	5·54	6·73	0	0·782
m.15699G>C	03 - B	40·18	80%	14·34	13·25	15·98	56·42	99·32	0·68	0	0	6·4	8·37	1·06	0·867
	03 - 12 W	30·39	86%	20·54	8·63	9·97	60·85	100	0	0	0	5·12	7·17	0·37	0·772
m.3250T>C	05 - B	238·46	97%	0	0	0	100	100	0	0	0	2·39	54·19	0	0·775
	05 - 12 W	158·88	97%	0	0	0·11	99·89	99·89	0	0	0·11	6·74	43.00	0	0·833
m.5690A>G	06 - B	52·39	45%	26·19	34·52	14·29	25	63·1	29·76	3·57	3·57	4·55	4·55	2·27	0·816
	06 - 12 W	72·34	42%	68·49	7·56	4·41	19·54	95·59	1·68	1·68	1·05	5·56	9·20	0	0·744
m.5703G>A	07 - B	28·36	46%	71·86	11·06	6·03	11·06	90·45	1·51	2·51	5·53	5·9	3·39	8·9	0·780
	07 - 12 W	23·13	38%	56·25	37·5	0	6·25	50	25	12·5	12·5	5·22	42·86	0	0·739
m.12271T>C	08 - B	23·38	15%	96·04	1·76	0·44	1·76	98·24	0	0	1·76	4·44	16·03	0	0·714
	08 - 12 W	48·97	39%	68·93	16·36	6·62	8·09	85·85	5·88	7·54	0·74	5·75	5·19	0	0·824
m.14709T>C	09 - B	31·13	69%	81·4	3·37	2·75	12·48	94·26	1	1·12	3·62	5·06	3·43	3·19	0·767
	09 - 12 W	46·68	65%	75·12	5·37	3·9	15·61	91·71	3·9	0	4·39	5·28	9·17	0	0·743
m.14709T>C	10 - B	39·55	69%	65·8	16·75	10·85	6·6	86·79	6·6	6·6	0	5·9	5·38	22·69	0·753
	10 - 12 W	39·06	73%	69·58	7·31	3·3	19·81	84·67	5·19	1·89	8·25	5·41	1·85	8·02	0·755
m.3243A>G	11 - B	38·20	72%	100	0	0	0	100	0	0	0	5·04	1·44	0·48	0·808
	11 - 12 W	55·93	72%	94·41	1·4	0·8	3·39	98·8	0·6	0·4	0·2	4·09	22·69	0	0·754

Individual patient muscle biopsy data at baseline and Week 12 for the Completers Analysis Set (N = 10). Only subjects with biopsies at both time points are included.
Abbreviations: B, Baseline; W, weeks; CI, Complex I, CIV, Complex IV, CPT1B, carnitine palmitoyltransferase 1B; Pos, Positive; Int, Intermediate; Neg, negative.

Muscle Biopsy – Individual Subject Results

mtDNA copy number and mutation load (heteroplasmy levels)
mtDNA copy number increased by subjects #02, 06, 08, 09 and 11 after 12 weeks mavodelpar (REN101) treatment (range 6% to 108%), while levels decreased in subjects #01, 03, 05 and 07 (range 17% to 33%). There was no change is subject #10.

Muscle heteroplasmy levels decreased in three subjects after mavodelpar treatment (#06, 07 and 09) and increased in five subjects (#01, 02, 03, 08 and 10). There was no change in #05 or #11 (**Table S9**).

OXPHOS protein level analysis
Analysis of OXPHOS protein levels was performed on all samples at Baseline and Week 12 (n = 10). Several subject biopsies at Baseline (#02, 07, 08, 10) and Week 12 (#01, 07, 10) had <300 analysable fibres and may therefore have higher variability between samples.[14] OXPHOS analysis classifies muscle fibres based on the intensity of immunofluorescence signal of complex I and complex IV relative to control muscle biopsies. These values are determined by calculating the distribution of intensity for controls, classifying muscle fibres based on which standard deviation they lie within. The classifications are as follows: positive fibres (between 0 and -3 SD difference from control), intermediate positive (between -3 and -4·5 SD difference from control), intermediate negative (between -4·5 and -6 SD difference from control and negative (-6 SD difference from control). The percentage of fibres in each of these groups is then calculated. A severely affected individual will typically have a high percentage of fibres classed as “negative”. See Rocha et al.[10] for a thorough explanation and examples of deficiencies.

Complex I
Complex I deficiency was the predominant type of deficiency seen in all subject biopsies (expect #11, Baseline) having complex I negative fibres (**Table S9**). The relationship between the proportion of complex I deficient fibres and treatment with REN001 is complex. Some subjects (#01, 03, 06, and 10) increased the proportion of complex I positive fibres, whereas the remaining subjects decreased the proportion after mavodelpar treatment. The proportion of negative fibres after treatment increased in #02, 03, 08, 09, 10 and 11 after treatment, with the remaining four subjects showing a decrease in the proportion of complex I negative fibres.

Complex IV (CIV)
In general, the biopsies showed little complex IV deficiency, indeed only #06, 07, 08, 09 and 10 had CIV negative fibres (**Table S9**). Only #06 increased the proportion of positive CIV fibres after mavodelpar treatment, with #07, 08, 09 and 10 all decreasing the proportion of positive fibres. Likewise, #06 and 08 decreased the proportion of CIV negative fibres after mavodelpar treatment, #07, 09 and 10 increased the proportion of CIV negative fibres after treatment (**Table S9**).

CPT1B expression analysis
CPT1B is the skeletal muscle isoform of Carnitine Palmitoyltransferase. This protein is responsible for the transport of fatty acids into mitochondria. To understand the changes in CPT1B levels associated with mavodelpar treatment, immunofluorescence was used to analyse the levels of CPT1B in skeletal muscle biopsies. The mitochondrial mass marker, Porin, was also analysed to enable a normalisation of CPT1B levels. The level of CTP1B in the subject biopsies was compared to unaffected controls by quantifying the proportion of fibres with levels of CPT1B higher than the 95% predictive interval for the controls (linear regression model). Therefore, the proportion of fibres with higher than expected levels of CPT1B is quantified. The level of CPT1B per fibre increased in #02, 06, 07, 09 and 11. The remaining subjects all showed a decrease in the proportion of fibres that exceed the controls 95% confidence interval after treatment.

ATP/ADP ratio
Measurement of the ADP/ATP provides an insight into the effectiveness of oxidative phosphorylation, with patients typically showing decreased ATP/ADP ratio during exercise (Reviewed in Kemp et al 2007[15]). The ATP/ADP ratio increased in #05, 06, 08, and 09 after mavodelpar treatment. The remaining subjects all showed a decrease in ATP/ADP ratio after mavodelpar treatment.

Muscle Biopsy –Transcriptomic Analysis

RNA alignment and quantification
FASTQ files for 14 skeletal muscle biopsy samples (n = 7 baseline and n = 7 following 12 weeks mavodelpar (REN101) treatment, below) were aligned to the human genome (Ensembl GRCh38[3]) using HISAT2 v2.1.0[4]. RNA transcripts were quantified using the Ensembl GRCh38 v101 cDNA reference and Salmon v1.3.0[5] using the 'selective alignment mode' option and correcting for sequence-specific (--seqBias), GC-content (--gcBias) and positio0nal biases (--posBias). Transcript counts were translated into gene-level counts using tximport v1.26.1[6] and annotated using the Ensembl v101 GRCh38 reference.

Table S10. RNA-seq sample metadata and pairing information

Sample ID	Age at biopsy	Sex	Pair	Baseline Seq ID	Week 12 treatment Seq ID
1130/1655	45	M	1	3-1130_S3	6-1655_S6
1005/1825	47	F	2	1-1005_S1	12-1825_S12
1635/1676	48	F	3	5-1635_S5	8-1676_S8
1092/1779	52	F	4	2-1092_S2	11-1779_S11
1295/1670	55	F	5	4-1295_S4	7-1670_S7
1838/1866	57	M	6	14-1838_S13	15-1866_S14
1733/1765	60	M	7	9-1733_S9	10-1765_S10

Sample quality control and filtering
Sample level quality control was assessed in both samples at baseline and after 12 weeks to identify sample failure, RNA contamination and abnormal global transcriptome issues. Fastq Screen v0.14.1[7] and FastQC v0.11.7 (https://www.bioinformatics.babraham.ac.uk/projects/fastqc/) were used to assess contamination and obtain general sequencing metrics. One sample (female, baseline, pair 2) failed QC and was removed from further analysis along with its pair (female, Week 12 treatment, pair 2) leaving a final dataset of 12 RNAseq samples (n = 6 baseline and n = 6 Week 12 mavodelpar treatment) in six pairs. Alignment metrics were obtained using *CollectRnaSeqMetrics* from Picard v2.27.5 (http://broadinstitute.github.io/picard/) and *stats* from SAMtools v1.6[8]. Principal component analysis (PCA) was used for data exploration and to assess confounders (**Fig S 1a**).

Differentially expressed genes (DEGs)
Normalization and differential expression analysis of genes was carried out using DESeq2 v1.38.2[9]. Gene raw counts were filtered for low counts (> 10 counts in the smallest sample group). Gene counts were library normalised using the default median-of-ratios method in DESeq2 (Anders and Huber, 2010). Differential expression between treated and untreated using a Wald test in DESeq2, adjusted for specific technical (e.g., mapped reads) library and biological covariates (e.g., sex) and 'sample' to account for baseline/Week 12 treatment pairing. Similar to previous work[10], significantly differentially expressed genes were defined as those that had an FDR < 0.05 (or unadjusted $p < 0.00003$) and a $\log_2$ fold change ($\log_2$FC) < −0.58/ > 0.58. All analysis was performed in R (v4.3).

Results
Transcriptomic analysis Identified 10 DEGs (**Fig S 1b**): *ABHD1*, *PREB*, *CPT1A*, *ST14*, *AQP9*, *MICU3*, *JAML*, *PKHD1L1*, *FAXC* and *C5* (Detailed in **Table S11**). Notably, *ABHD1*, *CPT1A* and *MICU3* are nuclear encoded mitochondrial proteins.

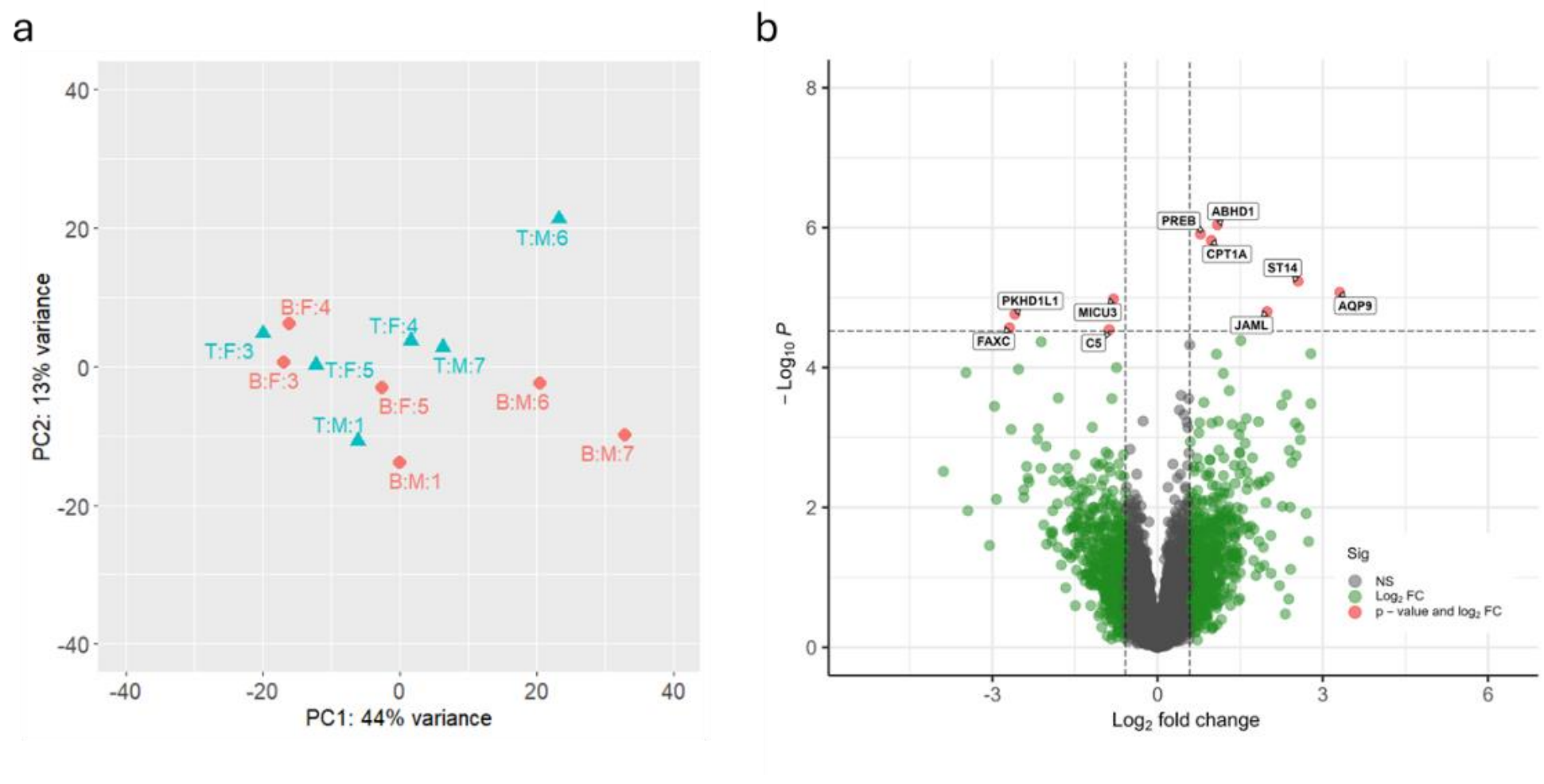

Figure S1. Transcriptomic Analysis
(**a**) PCA plot of RNAseq data showing 12 week mavodelpar treatment (n = 6, 'T' in blue triangles) and baseline (n = 6, 'B' in red circles) samples (M = male and F = female) and sample pairing (=1-6, table above). (**b)** Volcano plot showing differential expression between Week 12 mavodelpar treatment and baseline. Red dots indicate RNAs above the fold change and p value threshold (FDR < 0.05 (or unadjusted $p < 0.00003$) and a $\log_2$ fold change ($\log_2$FC) < −0.58/ > 0.58).

Table S11. Differentially Expressed Genes

Gene ID	Gene	Mitochondrial	Base Mean Exp	Log2FC	LFC SE	unadjusted p value	adjusted p value
ABHD1	Abhydrolase Domain Containing 1	**Yes**	195.47	1.09	0.22	9.1E-07	7.6E-03
PREB	Prolactin Regulatory Element Binding	No	2592.31	0.78	0.16	1.2E-06	7.6E-03
CPT1A	Carnitine Palmitoyltransferase 1A	**Yes**	970.36	0.98	0.20	1.5E-06	7.6E-03
ST14	ST14 Transmembrane Serine Protease Matriptas	No	24.07	2.55	0.56	5.8E-06	2.2E-02
AQP9	Aquaporin 9	No	62.13	3.31	0.74	8.4E-06	2.5E-02
MICU3	Mitochondrial Calcium Uptake Family Member 3	**Yes**	144.22	-0.80	0.18	1.0E-05	2.6E-02
JAML	Junction Adhesion Molecule Like	No	152.12	1.99	0.46	1.6E-05	3.3E-02
PKHD1L1	Polycystic Kidney And Hepatic Disease-Like 1	No	81.89	-2.59	0.60	1.7E-05	3.3E-02
FAXC	ailed Axon Connections Homolog	No	73.36	-2.69	0.64	2.7E-05	4.3E-02
C5	Complement C5	No	61.99	-0.88	0.21	2.9E-05	4.3E-02

Table showing differentially expressed genes between Week 12 mavodelpar treatment and baseline, n = 6 patients (above the fold change and *p* value threshold (FDR < 0.05 (or unadjusted $p < 0.00003$) and a $\log_2$ fold change ($\log_2$FC) < −0.58/ > 0.58). Shown is Gene ID, gene name, base mean expression, $\log_2$ fold change (Log$_2$FC), log fold change standard error (LFC SE), unadjusted *p* value and adjusted *p* value (FDR). Nuclear encoded mitochondrial proteins are highlighted (Mitochondrial) annotated according to MitoCarta 3.0.

Gene Ontology (GO) and pathway analysis

Enrichment of gene sets was performed using FGSEA (v3.21). Gene set enrichment analysis (GSEA)[11] was based on gene lists ranked by fold change. All genes included in differential expression testing were used as the background set. Gene sets were obtained as Gene Ontologies[12] (including biological processes, molecular functions, and cellular compartments) using Hallmark Gene set h.all.v2023.2.Hs.symbols.gmt.

Results

To gain an overview of global alterations in gene expression patterns we performed gene set enrichment analysis (GSEA)[11]. After classifying into GO terms, we identified 16 biological processes that were significantly enriched in 12 week mavodelpar treatment (versus baseline) samples (adjusted $p < 0.05$, **Table S12**). The majority (13/16) were upregulated in 12 week mavodelpar treatment compared to baseline. Notably, fatty acid metabolism (*CPT1A*), adipogenesis (*PREB*), P53 (*ST14*) and inflammatory response pathways (*AQP9* and *C5*) were all elevated in 12 week mavodelpar treatment samples compared to baseline.

Table S12. Gene Set Enrichment Analysis

Hallmark Pathway	p value	adjusted p value	Log2 Err	ES	NES	Size
COMPLEMENT	1.1E-06	1.9E-05	0.6436	0.4203	1.9292	169
EPITHELIAL MESENCHYMAL TRANSITION	8.6E-07	1.9E-05	0.6594	-0.4007	-1.9358	185
INFLAMMATORY RESPONSE	6.6E-07	1.9E-05	0.6594	0.4211	1.9199	161
ALLOGRAFT REJECTION	2.0E-06	2.5E-05	0.6273	0.4210	1.9116	155
BILE ACID METABOLISM	5.8E-04	4.8E-03	0.4773	0.4327	1.8027	86
MYC TARGETS V1	5.2E-04	4.8E-03	0.4773	0.3363	1.5756	195
MTORC1 SIGNALING	3.3E-03	2.4E-02	0.4317	0.3092	1.4493	198
ADIPOGENESIS	4.2E-03	2.5E-02	0.4070	0.3090	1.4514	197
XENOBIOTIC METABOLISM	4.6E-03	2.5E-02	0.4070	0.3272	1.4911	160
FATTY ACID METABOLISM	7.3E-03	3.3E-02	0.4070	0.3255	1.4638	145
MITOTIC SPINDLE	6.7E-03	3.3E-02	0.4070	-0.2985	-1.4460	192
ESTROGEN RESPONSE EARLY	1.0E-02	4.2E-02	0.3807	-0.2980	-1.4244	172
IL6 JAK STAT3 SIGNALING	1.4E-02	4.7E-02	0.3807	0.3995	1.6004	70
KRAS SIGNALING UP	1.5E-02	4.7E-02	0.3807	0.2998	1.3763	169
P53 PATHWAY	1.5E-02	4.7E-02	0.3807	0.3070	1.4234	181
PEROXISOME	1.3E-02	4.7E-02	0.3807	0.3552	1.4860	90

Results of Gene set enrichment analysis (GSEA) of genes (based on ranked fold changes) grouped into Hallmark pathways. Shown is the name of the pathway, enrichment significance (unadjusted and adjusted *p* values), the enrichment score (ES), normalized enrichment score (NES), and Log2 err refers to log2 of the standard error of the Normalized Enrichment Score (NES).

Table S13. Modified Fatigue Impact

	Full Analysis Set (N = 23)										Completers Analysis Set[a] (N = 17)				
	Baseline		Week 1		Week 2		Week 4		Week 8		Baseline		Week 12	Change from Baseline	*P* value
	n	Mean ± SD	n	Mean ± SD	n	Mean ± SD	n	Mean ± SD	n	Mean ± SD	n	Mean ± SD	Mean ± SD	Mean ± SD (95% CI)	
MFIS total score (0-84)[b]	23	46·5 ± 17·5	23	41·6 ± 17·0	23	39·3 ± 19·7	18	39·8 ± 16.0	18	39·5 ± 18·9	17	50·4 ± 18·6	39·9 ± 19·1	-10·5 ± 11·4 (-16·3 to -4·6)	**0·004**
Physical subscale (0-36)	23	24·0 ± 7·7	23	22·3 ± 7·4	23	21·1 ± 8·7	18	21·2 ± 7·4	18	20·9 ± 9·1	17	25·7 ± 7·8	20·6 ± 8·6	-5·1 ± 6·3 (-8·3 to -1·8)	**0·008**
Cognitive subscale (0-40)	23	18·4 ± 9.0	23	15·5 ± 9·3	23	14·7 ± 10·4	18	14·7 ± 9·1	18	15·1 ± 9·7	17	20·1 ± 9·6	15·2 ± 9·9	-4·9 ± 4·8 (-7·3 to -2·4)	**0·004**
Psychosocial subscale (0-8)	23	4·1 ± 2·1	23	3·8 ± 2·1	23	3·5 ± 2·0	18	3·8 ± 1·9	18	3·5 ± 2·3	17	4·5 ± 2·1	4·0 ± 2·3	-0·5 ± 1·6 (-1·3 to 0·3)	0·19

Abbreviations: MFIS, Modified Fatigue Impact Scale.

a. Completers Analysis Set: those subjects who completed Part A; the 12 week treatment phase. Only those subjects with data at both Baseline and Week 12 are included in summaries.
b. Total MFIS score is computed by adding scores on the physical (9-items), cognitive (10-items), and psychosocial (2-items) subscales. Higher scores indicate a greater impact of fatigue on a person’s activities.

Table S14. Brief Pain Inventory (BPI) – (Short Form)

	Full Analysis Set (N = 23)										Completers Analysis Set[c] (N = 17)				
	Baseline		Week 1		Week 2		Week 4		Week 8		Baseline		Week 12	Change from Baseline	*P* value
	n	Mean ± SD	n	Mean ± SD	n	Mean ± SD	n	Mean ± SD	n	Mean ± SD	n	Mean ± SD	Mean ± SD	Mean ± SD (95% CI)	
**Pain Severity (0-10)[a]**	22	3·15 ± 2·39	23	2·58 ± 2.44	23	2·53 ± 2.48	18	3·29 ± 2·16	18	3·48 ± 2·48	17	3·72 ± 2·37	2·91 ± 2·23	-0·81 ±1·46 (-1·56 to -0·06)	**0·01**
Worst (0-10)	22	4·23 ± 3·32	23	3·91 ± 3·42	23	3·35 ± 3·23	18	4·94 ± 3·24	18	5·11 ± 3·16	17	4·88 ± 3·31	4.00 ± 3·06	-0·88 ± 2·42 (-2·13 to 0·36)	0·34
Least (0-10)	22	1·73 ± 1·70	23	1·43 ± 1·88	23	1·65 ± 1·97	18	1·78 ± 1·63	18	2·28 ± 2·14	17	2·12 ± 1·73	1·88 ± 1·58	-0·24 ±1·15 (-0·83 to 0·35)	0·54
Average (0-10)	22	3·91 ± 2·69	22	3·00 ± 2·60	23	2·91 ± 2·63	18	3·78 ± 2·44	18	3·89 ± 2·37	17	4·53 ± 2·62	3·12 ± 2·57	-1·41 ± 1·84 (-2·36 to -0·47)	0·08
Right Now (0-10)	22	2·68 ± 2·51	23	2·04 ± 2·44	23	2·13 ± 2·62	18	2·61 ± 2·30	18	2·56 ± 2·96	17	3·29 ± 2·54	2·53 ± 2·37	-0·76 ± 1·56 (-1·57 to -0·04)	0·21
**Pain Interference (0-10)[b]**	22	2·53 ± 2·71	22	2·30 ± 2·55	22	2·48 ± 2·78	18	2·83 ± 2·36	18	3·24 ± 2·62	17	3·18 ± 2·75	2·92 ± 2·57	-0·26 ± 1.63 (-1.10 to 0.58)	0·96
Relations with Others (0-10)	22	1·59 ± 2·36	22	1·45 ± 2·30	22	1·68 ± 2·42	18	1·83 ± 2·20	18	2·67 ± 2·72	17	2·00 ± 2·55	1·82 ± 2·27	-0·18 ± 1·38 (-0·89 to 0·53)	0·96
Enjoyment of Life (0-10)	22	2·27 ± 2·88	22	2·18 ± 2·94	22	2·41 ± 2·95	17	2·82 ± 2·72	18	2·89 ± 2·83	17	2·88 ± 3·02	2·82 ± 2·88	-0·06 ± 2·25 (-1·22 to 1·10)	1
Mood (0-10)	22	2·23 ± 2·69	22	1·59 ± 2·42	22	1·95 ± 2·57	18	2·11 ± 1·97	18	3·17 ± 2·79	17	2·76 ± 2·84	2·65 ± 2·85	-0·12 ± 2·57 (-1·44 to 1·20)	1
Sleep (0-10)	22	2·77 ± 2·91	22	2·36 ± 2·98	22	2·68 ± 3·26	18	3·56 ± 3·07	18	3·17 ± 2·71	17	3·47 ± 2·94	3·00 ± 2·89	-0·47 ± 1·46 (-1·22 to 0·28)	0·96
Walking Ability (0-10)	22	3·23 ± 3·42	22	3·27 ± 3·52	22	3·14 ± 3·17	17	3·35 ± 2·74	18	3·94 ± 3·40	17	4·06 ± 3·47	3·53 ± 3·12	-0·53 ± 2·10 (-1·61 to 0·55)	0·96
General Activity (0-10)	22	2·68 ± 2·87	22	2·82 ± 2·70	22	2·68 ± 3·05	18	2·94 ± 2·44	18	3·56 ± 2·53	17	3·41 ± 2·87	3·41 ± 2·94	0 ± 2·52 (-1·30 to 1·30)	1
Working (0-10)	22	3·00 ± 3·24	22	2·41 ± 2·87	22	2·82 ± 3·19	18	3·28 ± 2·82	18	3·28 ± 3·14	17	3·76 ± 3·31	3·29 ± 3·14	-0·47 ± 2·18 (-1·59 to 0·65)	0·96

N.B: Data analysis (after database lock) not specified in the statistical analysis plan included: Item scores for BPI Pain Severity (Worst, Least, Average, Right Now) and BPI Pain Interference (Relations with Others, Enjoyment of Life, Mood, Sleep, Walking Ability, General Activity, and Working).Completers Analysis Set: those subjects who completed Part A; the 12 week treatment phase. Only those subjects with data at both Baseline and Week 12 are included in summaries.

a. BPI – (Short Form) Pain Severity score is the average of the 4 pain items (a mean severity score). 0=no pain and 10=pain as bad as you can imagine.
b. BPI – (Short Form) Pain Interference score (impact of pain on functioning) is scored as the mean of the 7 interference items. 0=no interference and 10=interferes completely.
c. Completers Analysis Set: those subjects who completed Part A; the 12 week treatment phase. Only those subjects with data at both Baseline and Week 12 are included in summaries.

Supplementary References (Results)

1 Suomalainen A, Elo JM, Pietiläinen KH, Hakonen AH, Sevastianova K, Korpela M, et al. FGF-21 as a biomarker for muscle-manifesting mitochondrial respiratory chain deficiencies: a diagnostic study. Lancet Neurol. 2011 Sep;10(9):806-18.

2 Wiklund FE, Bennet AM, Magnusson PK, Eriksson UK, Lindmark F, Wu L, et al. Macrophage inhibitory cytokine-1 (MIC-1/GDF15): a new marker of all-cause mortality. Aging Cell. 2010 Dec;9(6):1057-64.

3 Cunningham F, Allen JE, Allen J, Alvarez-Jarreta J, Amode M R, Armean Irina M, et al. Ensembl 2022. Nucleic Acids Research. 2021;50(D1):D988-D95.

4 Kim D, Paggi JM, Park C, Bennett C, Salzberg SL. Graph-based genome alignment and genotyping with HISAT2 and HISAT-genotype. Nature Biotechnology. 2019 2019/08/01;37(8):907-15.

5 Patro R, Duggal G, Love MI, Irizarry RA, Kingsford C. Salmon provides fast and bias-aware quantification of transcript expression. Nature Methods. 2017 2017/04/01;14(4):417-9.

6 Soneson C, Love M, Robinson M. Differential analyses for RNA-seq: transcript-level estimates improve gene-level inferences [version 1; peer review: 2 approved]. F1000Research. 2015;4(1521).

7 Wingett SW, Andrews S. FastQ Screen: A tool for multi-genome mapping and quality control. F1000Res. 2018;7:1338.

8 Danecek P, Bonfield JK, Liddle J, Marshall J, Ohan V, Pollard MO, et al. Twelve years of SAMtools and BCFtools. GigaScience. 2021;10(2).

9 Love MI, Huber W, Anders S. Moderated estimation of fold change and dispersion for RNA-seq data with DESeq2. Genome Biol. 2014;15(12):550.

10 Craig DW, Hutchins E, Violich I, Alsop E, Gibbs JR, Levy S, et al. RNA sequencing of whole blood reveals early alterations in immune cells and gene expression in Parkinson's disease. Nat Aging. 2021 Aug;1(8):734-47.

11 Subramanian A, Tamayo P, Mootha VK, Mukherjee S, Ebert BL, Gillette MA, et al. Gene set enrichment analysis: a knowledge-based approach for interpreting genome-wide expression profiles. Proc Natl Acad Sci U S A. 2005 Oct 25;102(43):15545-50.

12 Ashburner M, Ball CA, Blake JA, Botstein D, Butler H, Cherry JM, et al. Gene ontology: tool for the unification of biology. The Gene Ontology Consortium. Nat Genet. 2000 May;25(1):25-9

Study Protocol Summary

1.1. Overall Study Design and Study Plan

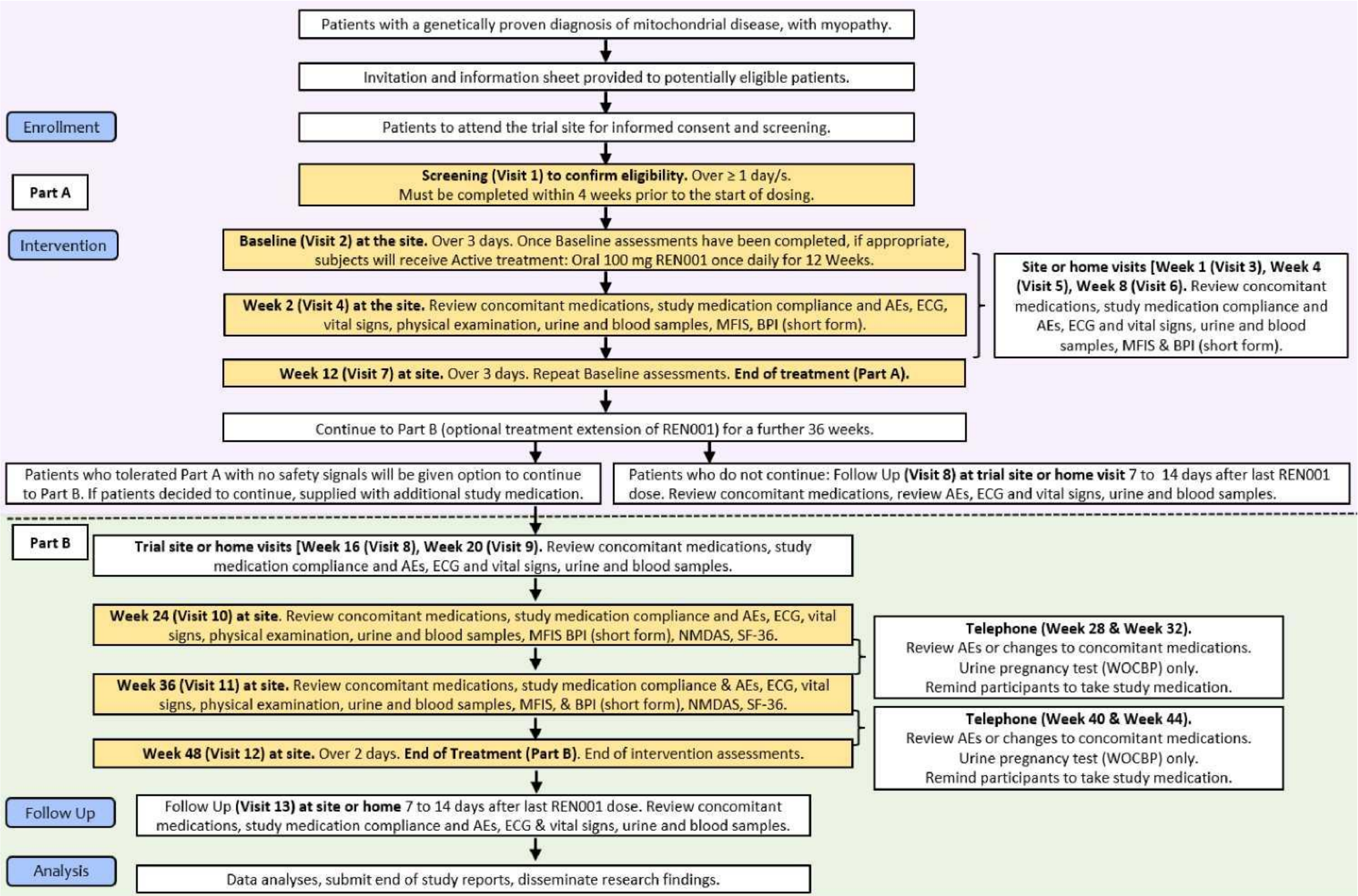

Figure S2. Study design and assessment schedule. Visits to the trial site are shown in yellow. Abbreviations: AEs, adverse events; BPI, Brief Pain Inventory; ECG, Electrocardiogram; MFIS, Modified Fatigue Impact Scale; NMDAS, Newcastle Mitochondrial Disease Adult Scale; SF-36, 36 Item Short Form Survey; WOCBP, women of childbearing potential

1.1.1 Inclusion and Exclusion Criteria

Inclusion criteria:

1. Males or females aged ≥16 years at Screening.
2. Established diagnosis of PMM according to the 2016 Rome Consensus recommendations[1]
3. A myopathy score of 2-4 on Question 5, Section III of the NMDAS.
4. Either confirmed m.3243A>G or other mtDNA pathogenic variant with myopathy.
5. No changes to exercise regimen within 30 days prior to Day 1 and willing to remain on the same exercise regimen for the duration of the trial.
6. Ambulatory and able to perform the study functional/exercise tests independently.
7. Able to remain on stable medication through the trial; must not commence or have changes in medication for diabetes.
8. Adequate kidney function defined as an estimated glomerular filtration rate (eGFR) ≥60 mL/min/1.73 m^2 using the Chronic Kidney Disease Epidemiology Collaboration (CKD-EPI) creatinine equation[2] at Screening and Baseline.
9. Able to swallow capsules.
10. Females: either of non-child-bearing potential or must agree to use a highly effective method of contraception from Screening. Men whose partners are women of childbearing potential (WOCBP) must also use contraception from Baseline.
11. Willing and able to give informed consent to participate in the trial.

Exclusion Criteria:

1. Participation in a prior REN001 (previously known as HPP-593) study.
2. Employee of the trial site or of the Sponsor's company or delegates.
3. Documented evidence of ongoing rhabdomyolysis.
4. Currently taking or anticipated to need a proliferator-activated receptor (PPAR) agonist during the study.
5. Motor abnormalities other than related to the mitochondrial disease that may interfere with the outcome measures.
6. Treatment with an investigational drug within 3 months prior to Day 1.
7. Currently taking or anticipated to need a prescription and/or non-prescription drug that may interfere with study endpoints.
8. Evidence of significant concomitant clinical disease that may need a change in management during the study or could interfere with the conduct or safety of this study [stable well-controlled chronic conditions such hypercholesterolemia, gastroesophageal reflux, or depression under control with medication (other than tricyclic antidepressants), are acceptable provided the symptoms and medications would not be predicted to compromise safety or interfere with the tests and interpretations of the study].
9. Diabetics who require treatment with insulin who are unwilling to undertake glucose monitoring for at least the first 12 weeks of dosing or in the Investigators opinion will not be able to adequately maintain glucose homeostasis.
10. History of cancer (excluding a history of in situ basal cell carcinoma in the skin).
11. Hospitalized within 3 months prior to Screening for any major medical condition [deemed by Principal Investigator (PI)].
12. Clinically significant cardiac disease or ECG abnormalities that in the opinion of the PI should exclude study enrolment.
13. Any condition possibly reducing drug absorption [e.g., gastrectomy or serious gastrointestinal dysmotility].
14. Poor nutritional status as determined by the Investigator.
15. History of clinically significant liver disease and/or liver function tests [Aspartate transaminase (AST), alanine aminotransferase (ALT) or total bilirubin (TB) >1·5 x upper limit of normal (ULN)]. Subjects with liver function tests (LFTs) >1.5 x ULN and <2 x ULN at Screening may be retested once. Subjects with an isolated elevated bilirubin (e.g., <2 x ULN) may be included if the cause is due to a benign hereditary disorder of metabolism such as Gilbert's syndrome.
16. Evidence of clinically significant muscle damage tests [creatine kinase (CK) >3 x ULN]. Subjects with CK >3 x ULN and <5 x ULN at Screening may be retested once.
17. Positive hepatitis B surface antigen (HBsAg), hepatitis B core antibody (HBcAb), hepatitis C or human immunodeficiency virus (Screening).
18. Pregnant or nursing females.
19. History of sensitivity to PPAR agonists.
20. Anticoagulant treatments that would preclude muscle biopsy.
21. Donation or intent to donate blood, or blood components during the study or within 1 month after completion of the study.
22. History of drug abuse or positive urine drug screen at Screening. Positive results for opiates and/or benzodiazepines will only be allowed if supported by a corresponding prescription from a medically qualified person.
23. History of regular alcohol consumption exceeding 14 units/week (1 unit = 75 mL of wine or 250 mL of beer or 25 mL of spirits) within 6 months of Screening.
24. Inability to comprehend or unwilling to follow the study requirements including restrictions on treatments, attendance at clinic visits, completion of questionnaires and participation in laboratory testing as called for by the protocol.
25. Any other severe acute or chronic medical or psychiatric condition or laboratory abnormality that may increase the risk associated with study participation or investigational product administration or may interfere with the interpretation of study results and, in the judgment of the Investigator in discussion with the Medical Monitor, would make the subject inappropriate for entry into this study.
26. Subjects who are not eligible or have a contraindication for cataract surgery.

1.1.2 Trial Endpoints and Outcome Measures

Primary Endpoint
The primary outcome is the safety and tolerability of mavodelpar (REN001), evaluated by the number and severity of adverse events (AEs) at the end of the 12 week intervention period. AEs will be reported continuously during the study and reviewed at each study visit.

Secondary Endpoints:
Safety: Absolute values, changes from Baseline (at 12 and 48 weeks if applicable) and incidence of clinically significant changes in:

a) Laboratory safety tests (haematology and biochemistry). Blood and urine samples will be collected at Screening, Baseline, Weeks 1, 2, 4, 8, 12, 16, 20, 24, 36 and 48 (Weeks 16 to 48 only if subject participates in Part B) and Follow-up (see **Table S17** for parameters to be tested).
b) Electrocardiogram (ECG) measurements will be taken using a 12-lead ECG machine and following ≥10 minutes rest in a supine position. ECGs will be recorded at: Screening, Baseline and Week 12 pre-dose and 4 hours post-dose, Weeks 1, 2, 4 and 8, Weeks 16, 20, 24, 36 and 48 (if subject participates in Part B) and Follow-up.
c) Supine vital signs. BP (systolic and diastolic, mmHg) and pulse in the brachial/radial artery (beats per minute) will be measured using the subject's dominant arm throughout the trial at: Screening, Baseline and Week 12 (pre-dose and 4 hours post-dose), Weeks 1, 2, 4 and 8, Weeks 16, 20, 24, 36 and 48 (if subject participates in Part B, Follow-up). Subjects should be supine for ≥5 minutes before taking measurements.

Pharmacokinetic Endpoints
REN001 plasma concentrations using population pharmacokinetic methodology:

a) Pharmacokinetic blood samples for determination of REN001 concentrations will be collected via direct venepuncture or indwelling cannula. Samples will be taken at Baseline and Week 12 [pre-dose and at 1, 2 and 4 hours post-dose, and immediately prior to discharge (unless the subject leaves less than 2 hours after the 4 hour post-dose sample)]. Samples will also be taken at 1, 2, 4 and 8 weeks post-treatment.
b) Identification of metabolites using pooled plasma. A separate blood sample will be collected during Week 12 for metabolite identification.

Pharmacodynamic Endpoints

a) Absolute values and changes from Baseline in serum biomarkers: fibroblast growth factor 21 (FGF-21) and growth differentiation factor 15 (GDF-15) levels after 12 weeks. Serum samples may also be used to analyse markers of inflammation and muscle injury (this decision will be made upon review of safety data).
b) Absolute values and changes from Baseline in acylcarnitine panel (pre-dose) after 12 weeks. Fasting blood samples will be collected via a blood spot (either ear lobe or finger prick) onto a specific sample card.
c) Changes from Baseline in muscle histopathology after 12 weeks, including Haematoxylin and Eosin staining (general tissue architecture); modified Gomori Trichrome ("ragged-red" muscle fibres)[3] typical in mitochondrial myopathy; succinate dehydrogenase (SDH) and cytochrome c oxidase (COX) activities.

Exploratory Endpoints
Predefined exploratory efficacy evaluations were categorized into changes from Baseline in:

a) Exercise capacity, as measured by peak and constant work rate sub-maximal exercise tests, and functional assessments including the 12-minute walk test (12MWT) and 30 second sit-to-stand (STS) at 12 and 48 weeks post-treatment (if optional extension, Part B is completed).
b) Disease severity as quantified by Newcastle Mitochondrial Disease Adult Scale (NMDAS) and quality of life evaluated using version 1 of the RAND 36-Item Short Form Health Survey (SF-36) at 12, 24, 36 and 48 weeks post-treatment (if optional extension, Part B is completed).
c) Perceived fatigue impact gauged by the Modified Fatigue Impact Scale (MFIS) and pain experience rated on the short form of the Brief Pain Inventory (BPI) at 1, 2, 4, 8, 12, 24, 36 and 48 weeks post-treatment (if optional extension, Part B is completed) and
d) Muscle biopsy analyses at Baseline and Week 12.

Measures of Exercise Capacity

- **Peak (Symptom-limited) Cardiopulmonary Exercise Test** was performed using an electronically braked recumbent cycle ergometer to assess changes in peak oxygen consumption (VO_{2peak}), maximal power output (watts), anaerobic threshold and oxygen extraction, using an electronically braked recumbent cycle ergometer via a ramp protocol, with resistance consistently increased at 5 to 15 watts/minute. Heart rate [12-lead ECG], gas exchange, ventilation, stroke volume and cardiac output (via bioreactance) was continuously monitored.

  Blood lactate (sampled via the ear or finger), Rate of Perceived Exertion (Borg, 6-20) and BP were measured every 2 minutes and/or immediately upon exhaustion.
- **Sub-maximal Exercise Test** was performed at a constant workload corresponding to 50 - 60% of peak

exercise capacity (VO_{2peak}) (calculated during the peak exercise test) for 30 minutes or until exhaustion. Heart rate [12-lead ECG], gas exchange, ventilation, stroke volume and cardiac output (via bioreactance) were continuously monitored. Blood lactate (sampled via the ear or finger), Rate of Perceived Exertion scale (Borg, 6-20) and BP will be measured every 10 minutes and/or immediately upon exhaustion. Time to exhaustion was recorded.

Measures of Functional Capacity

- **12-minute walk test (12MWT)** evaluates exercise tolerance (performance-based walking capacity test). Participants were instructed to cover as much ground as possible during the time period, at a self-determined walking speed, pausing to rest when needed. Due to the availability of resources at the two study sites, the test took place in either an enclosed quiet space - a continuous 25 m oval track (Newcastle) or a straight 10 m track in a corridor (London). All participants walked along a flat, hard surface with positive verbal encouragement at approximately minute intervals. The total distance walked in 12 minutes was recorded. Rating scale of perceived exertion (RPE) (Borg, 6 to 20), HR and BP was obtained before and immediately after the test.
- **30 second sit-to-stand (STS) test** was used as a proxy evaluation of functional lower extremity strength. The STS has high test-retest and inter-rater reliability [intraclass correlation coefficient (ICC) = 0.89] and was performed in accordance with its original protocol.[4] Following a practice trial, participants were instructed to fully rise and sit down as quickly as possible, while keeping their arms folded across their chest. The maximal number of full stands completed in 30 seconds was recorded.

Measures of Disease Severity

- **The Newcastle Mitochondrial Disease Adult Scale (NMDAS)** is a semi-quantitative clinical rating scale designed specifically to assess disease burden for all forms of adult mitochondrial disease.[5]

Measures of Quality of Life

- **36-Item Short Form Health Survey (SF-36)** (Version 1.0 developed by RAND, as part of the Medical Outcomes Study) measures self-reported health-related QoL (HRQoL). It comprises 36 items which cover eight health domains: physical functioning, bodily pain, role limitations due to physical health problems, role limitations due to emotional problems, energy/fatigue, emotional well-being, social functioning, bodily pain and general health. It also includes a single item that provides an indication of perceived change in health. Scores for each subscale range from 0 to 100, with a higher score indicating a better health state. The SF-36 is well used and is considered valid and reliable.[6]

Measures of Perceived Fatigue Impact

- **Modified Fatigue Impact Scale (MFIS)** is a 21-item shortened version of the 40-item Fatigue Impact Scale (FIS),[7] designed to assess the perceived effect of fatigue on three domains of daily life, physical (11 items), cognitive, (10 items), and psychosocial functioning (two items). The maximum total sum score is 84, with higher scores indicating a greater impact on QoL.

Measures of Pain Experience

- **Brief Pain Inventory (Short Form)** is a widely used self-reported measure of pain which assesses two outcome domains. (1). pain intensity [i.e. severity, singly represented by pain at its "worst", "least", "average" and "right now" and by a composite of the four pain items (mean severity score)] and (2) the impact of pain on daily functioning (i.e. interference, typically scored as a mean of how pain has interfered with seven daily activities).

Muscle Biopsy Analyses

- **mitochondrial DNA copy number**. A previously described Taqman qPCR assay[8] was used to compare the number of copies of the mitochondrial gene *MTND1*, to the nuclear encoded gene *18S rRNA* in homogenate skeletal muscle. *MTND1* was amplified using the primers: 5'-CCCTAAAACCCGCCACATCT-3' and 5'-GAGCGATGGTGAGAGCTAAGGT-3'. A fluorescent probe: 6FAM-5-'CCATCACCCTCTACATCACCGCCC-3'- TAMRA was used to detect amplification of *MTND1. 18S rRNA* was amplified using the primer pairs: 5'-GCCGCTAGAGGTGAAATTCTTG-3' and 5'-CATTCTTGGCAAATGCTTTCG-3'. A fluorescent probe: 6FAM-5'CCGGCGCAAGACGGACCAGA3'-

  TAMRA was used to detect gene amplification. The ratio of the two genes was used to calculate mitochondrial DNA copy number.
- **Heteroplasmy level (muscle)** was quantified using a pyrosequencing based assay as previously described.[9] A region flanking the mutation was amplified using PCR. The PCR product was subsequently analysed using a PyroMark Q24 pyrosequencer using primers specific to each variant.
- **Mitochondrial oxidative phosphorylation (OXPHOS) protein expression**. Mitochondrial OXPHOS protein abundance (complex I and complex IV) and a mitochondrial mass marker (porin) was quantified in individual myofibres via quadruple immunofluorescence (assay adapted from Rocha et al., WCMR[10]). Ten µm muscle sections from controls and patients were fixed with paraformaldehyde, permeabilized with methanol and blocked with

normal goat serum before primary antibodies specific to the proteins of interest (NDUFB8 for complex I, COX1 for complex IV, VDAC1/porin for mitochondrial mass and laminin for fiber membranes, or a corresponding no primary control), and secondary antibodies, conjugated to specific fluorophores, were added. Analysis firstly involves segmenting each individual fiber using immunoassay analyser software and extracting the raw fluorescent intensity per fiber for each channel from both non- mitochondrial control muscle and patient sections. Data generated was statistically analysed using a custom- built R script, corrected for background in each channel. Fibres were classified as being positive, intermediate positive, intermediate negative, or negative for complex I or complex IV, based on Z-scores calculated from a control population.[10] The activities of complex IV and complex II were analysed using sequential COX/SDH histochemistry.[10]

- **Carnitine Palmitoyltransferase 1B (CPT1B)** is an enzyme involved in fatty acid beta-oxidation in muscle mitochondria. CPT1B protein expression was quantified via immunofluorescence (assay adapted from Rocha et al., WCMR[10]). Control and patient sections were processed as above and stained with antibodies against specific proteins of interest; CPT1B, VDAC1/porin (mass marker), and Laminin (fiber membrane marker). Analysis was carried out as above and data was analysed using a custom-built R script which will carry out background correction and normalization. The percentage of patient fibres with CPT1B levels above or below a wild-type predicted interval was calculated at Baseline and after treatment. To account for mitochondrial mass, a linear regression model was generated between values observed for CPT1B and VDAC1 in all controls and the 95% predictive interval around the regression displayed. Fibres were classed as deficient for CPT1B if outside the 95% predictive interval for control fibres.
- **ATP and ADP levels.** As OXPHOS is the major source of cellular ATP, a luminescence-based assay was employed to measure skeletal muscle ATP/ADP ratio as a surrogate of OXPHOS function (assay adapted from the Murphy lab, MRC Mitochondrial Biology Unit, Cambridge) (Porteous et al.,1998).[11] ATP and ADP are extracted from snap frozen tissue samples using perchloric acid and the extract is then neutralized with potassium hydroxide. The bioluminescence reaction[12] produces photons in proportion to ATP, luciferin and luciferase concentrations, allowing the ATP in the samples to be determined against ATP standards by measuring light production in a luminometer. ADP is measured by first degrading the endogenous ATP with ATP sulfurylase and then enzymatically converting ADP to ATP using a pyruvate kinase/phospho(enol)pyruvate cocktail.
- **mRNA transcriptomics.** Where sufficient biopsy material allowed, transcriptomic analysis was performed to compare Baseline and 12 week gene expression levels. Total RNA was extracted from muscle samples using the Qiagen miRNeasy kit according to manufacturer's instructions. RNA integrity and quantity was assessed on an Agilent Tapestation system (minimum requirements: RIN >7, >2000ng RNA and [RNA] >50 ng/µl). mRNA libraries were created using the Illumina TruSeq Stranded mRNA kit and sequenced on the Illumina NextSeq 500 platform following the manufacturer's protocols. Quality checks of raw RNASeq data were made using FastQC (v.0.11.9), FastQ screen (v0.14.1); the quality of the aligned data was checked using alignment statistics from HISAT2 (v2.2.1), feature. Counts (v2.0.0) and QualiMap (v2.2.1); the threshold for data quality were met. The counts estimates for the transcripts were obtained using Salmon (v1.3.0) with sequence, GC and positional bias corrections. The genome version used was Homo sapiens GRCh38; the quantification was done using only coding transcripts. The counts were summarized on gene level using tximport (v.3.13). DeSEQ2 (v.1.30.1) was used for differential expression (DE) modelling. The DE modelling was performed with minimum effect size specified as fold change of two and at FDR of 0.05.

Table S15. Trial Outcome Measurement Schedule

Visit	Screening [m]	Baseline	Wk 1	Wk 2	Wk 4	Wk 8	Wk 12 /Early Termination (Part A) [n]	Wk 16	Wk 20	Wk 24	Wk 28 & 32	Wk 36	Wk 40 & 44	Wk 48/ Early Termination (Part B) [o]	Follow Up [p, q]
**Days**		-2 to 1	7	14	28	56	82-84	112	140	168	196 & 224	252	280 & 308	335-336	
**Visit number**	1	2	3	4	5	6	7	8	9	10		11		12	
**Windows (days)**			±2	±3	±3	±3	±3	±5	±5	±5		±5		±5	
Informed Consent	X														
Demographics	X														
Medical/medication/drug/alcohol history [a]	X	X					X							X	
Concomitant medication review [b]		X	X	X	X	X	X	X	X	X	X	X	X	X	X
Physical examination, height, weight, [c]	X	X		X			X			X		X		X	
Supine vital signs [d]	X	X	X	X	X	X	X	X	X	X		X		X	X
Ophthalmic examination [e]	X [e]						X			X		X		X	
Pregnancy test (WOCBP only) [f]	X	X			X	X	X	X	X	X	X	X	X	X	X
Serum FSH (post-menopausal females)	X														
Serum HbA1c, [g]	X	X	X	X	X	X	X								
Blood for hepatitis B/C /HIV, [h]	X														
Blood sample for PK analysis, [i]		X													
Blood sample for metabolite identification [j]							X								
Drug and alcohol screen, [k]	X	X													
Safety laboratory tests (and urinalysis) [l]	X	X	X	X	X	X	X	X	X	X		X		X	X
Serum biomarkers (FGF-21, GDF-15) and acylcarnitine panel		X					X								
Muscle biopsy		X					X								
Exercise tests		X					X							X	
12-minute walk, 30-second STS tests		X					X							X	
NMDAS and SF-36		X					X			X		X		X	
MFIS and Brief Pain Inventory (Short Form)		X	X	X	X	X	X			X		X		X	
Dosing		X --- --- --- --------- -- -------- X						X-- ---- ------------ ----- ----- X							
PTE/AE collection and reporting	X	X 1 ---- x-- ----x- X 1 ----x- X						X 1 X ----- x ------- x X 1 ------ x-- X							X

Abbreviations: AE, adverse event; FSH, follicle-stimulating hormone; HbA1c, glycosylated haemoglobin; HIV, human immunodeficiency virus; MFIS, Modified Fatigue Impact Scale; NMDAS, Newcastle Mitochondrial Disease Adult Scale PK, pharmacokinetic; PTE, Pre-treatment events; SF-36, 36-Item Short Form Health Survey; STS, sit-to-stand; Wk, week; WOCBP, women of childbearing potential; FGF-21, fibroblast growth factor 21; GDF-15, growth differentiation factor 15.

[a] Participants will complete a medical history, including prescription and non-prescription drugs/treatments, topical products, vitamins, and dietary supplements taken in the last 4 weeks, exercise regimen, alcohol, drugs of abuse and tobacco use. At Week 48, this will be a review of changes in the participant medical history since Week 12

[b] At Screening, subjects will be asked what medications they are currently taking and at each subsequent study visit, will be asked what medications they have taken since the last study visit. Any medication taken after the first dose of study medication will be considered a concomitant medication. See **Table S16** for list of medications and treatments that participants must withhold within the indicated time interval prior to Day 1 and for the duration of the study, where possible until completion of the Follow-up visit.

[c] All physical examinations will be carried out by a qualified doctor. A full physical examination includes an assessment of head, neck, heart, lungs, abdomen, skin (including hair and nails), peripheral circulation, joints, general appearance, neurological examination. A brief physical examination includes the general appearance, heart, lungs, skin and a neurological examination. Participants will have a full physical examination, height and weight (Screening); full physical examination and weight (Baseline); brief, symptom-directed physical examination and weight (Weeks 2, 12 and 24, 36 and 48 (if continue into Part B).

[d] (BP, pulse rate, otic body temperature); 12-lead ECG measured after at least 5 minutes of supine rest. BP will be measured in the dominant arm (throughout the study).

[e] Can be performed at +/- 7 days of the visit day for scheduling reasons; conducted at Screening or Baseline (Pre-dose).

[f] Serum pregnancy testing will be completed at Screening; all other scheduled assessments will be urine pregnancy testing.

[g] HbA1c completed at Week 12 or the Early Termination visit if prior to Week 12.

[h] Serology markers: HBsAg, HBcAb, anti-hepatitis C virus serology, and HIV.

[i] On Day 1 (Baseline) and Day 84 (Week 12) of dosing, samples will be taken pre-dose and at 1, 2 and 4 hours post-dose, and just before the subject exits the trial site (unless the exit is less than 2 hours after the 4 hour draw). On other visits, samples can be taken at any time post-dose.

[j] Samples will be pooled from all subjects for analysis.

[k] Urine drug screen and urine or breath alcohol test. Subjects will not be withdrawn if test positive, but results may be used in the analysis of study data. See **Table S17** for list of drugs of abuse.

[l] See **Table S17** for list of Safety Laboratory Parameters.

[m] Screening visit to confirm eligibility must be completed within 4 weeks prior to the start of dosing.

[n] This visit will take place as soon as possible after the subject stops taking study drug.

[o] If any participant terminates during Part B, observations and assessments at Week 48 (Visit 12) will be completed as the Early Termination Visit.

[p] 7-14 days post-last dose of study drug. Subjects who prematurely discontinued from the study will also return for the Follow-up visit unless their Early Termination visit is >7 days after their last dose (Follow-up visit is not required). If the subject withdraws consent for continuing in the study, no further assessments will be performed.

[q] The maximum study duration for participants included in Part A is 18 weeks (4 weeks screening, 12 weeks treatment and 2 weeks Follow-up) or 16 weeks where subject entered the optional extension. The planned maximum study duration for participants included in Parts A and B is 54 weeks (48 weeks treatment).

NB: Ophthalmic examinations were added to the study as a precautionary, urgent safety measure on 1st August 2019 due to an observation in a rat 26 week toxicology study (see Section 1.4 Risk-Benefit Assessment of the full Clinical Protocol) for details. All ophthalmic examinations will be carried out by a qualified person and will include measurements of eye pressure (tonometry), visual acuity, a slit-lamp examination and retinal examination (ophthalmoscopy). For scheduling reasons, the above assessments may be conducted +/- 7 days of the visit window given for the other assessments performed at the visit. For all ongoing subjects the above assessments should be conducted at the subject's next study visit (if this is scheduled to be a home visit it is to be changed to a study site instead) and subsequently at the time points listed above dependent on where the subject is in the study. In addition, any subject who experiences any ophthalmic symptoms should have an additional ophthalmic examination as appropriate.
As such, an exclusion criterion was added to exclude subjects who are not eligible or have a contraindication for cataract surgery [page 4]).

Table S16. Prohibited Medications

Medication	Time Interval Prior to Day 1
Statins (e.g., lovastatin, atorvastatin) *	1 week
Niacin	1 week
Anticoagulants	1 week
Bezafibrate and other PPAR agonists*	4 weeks
Cyclosporine and other immunosuppressive drugs	4 weeks
Oral or systemic steroids	4 weeks
Investigational drugs	3 months

*Following completion of Part A, subjects may be allowed to commence treatment with statins, bezafibrate and other PPAR agonists, if it is considered medically appropriate, following discussion with the Medical Monitor and Reneo Pharmaceuticals, Inc. Chief Medical Officer. Subjects who are receiving prohibited medications will suspend the medications and undergo washout during the screening period (appropriate washout periods in **Table S16**). Subjects may be re-screened once if the washout of current medications is required longer than 4 weeks or if LFT or CK levels are raised at initial screening.

Table S17. Safety Laboratory Parameters

<table>
<tr><td>Biochemistry
Sodium
Potassium
Calcium
Urea
Uric acid
C-reactive protein
Creatinine
Creatine kinase (CK)
Glucose (non-fasted)
Triglycerides
Troponin I
Total bilirubin (TB)
Total protein
Lipase
Albumin
Aldolase
Alkaline phosphatase (AP)
Aspartate aminotransferase (AST)
Alanine aminotransferase (ALT)
Gamma glutamyl transferase (GGT)
Total cholesterol
FSH (at Screening, post-menopausal females only)
Human chorionic gonadotropin (hCG) (at Screening, WOCBP only)</td><td>Haematology
Red cell count
Reticulocytes
Haemoglobin
Packed cell volume (haematocrit) Mean corpuscular volume (MCV) Platelet count
White cell count
Neutrophils
Lymphocytes
Monocytes
Eosinophils
Basophils</td></tr>
<tr><td colspan="2">Serological Markers (Screening only)
HIV
HBsAg
HBcAb
Anti-hepatitis C virus serology (by multi-antigen enzyme immunoassay (EIA))
HbA1c –also to be measured at Week 12 or the Early Termination visit if prior to Week 12</td></tr>
<tr><td colspan="2">Urinalysis
pH, protein, glucose, ketone, and blood hCG (measured at the Study Site) at Baseline, Weeks 4, 8, 12, 16, 20, 24, 36, 48 and Followup; kits will be provided to the participants to test at home on Weeks 28, 32, 40 and 44 (WOCBP only)
Any significant abnormalities in the urinalysis will be investigated via microscopy.</td></tr>
<tr><td colspan="2">Drugs of Abuse
Cocaine
Cannabinoids
Opiates
Barbiturates
Benzodiazepines
Methadone
Amphetamines</td></tr>
</table>

1.1.3 Treatment Discontinuation (Individual Stopping Rules)

Participants will be discontinued from the study in the following circumstances:

1. Enrolment in other clinical studies involving investigational products or enrolment in other types of clinical research judged scientifically or medically incompatible with the trial.
2. Use of prohibited concomitant medications (see **Table S16**).
3. Major protocol deviation. The discovery after administration of the first dose of study medication that the participant failed to meet protocol entry criteria or did not adhere to protocol requirements, and continued participation poses an unacceptable risk to the participant's health.
4. Lost to follow-up (subject does not return to the trial site and attempts to contact the subject is unsuccessful).
5. Sponsor decision to stop the study or to stop participation in the study for medical, safety, pharmacokinetic or regulatory, or other reasons consistent with applicable laws, regulations, and GCP.
6. Dose limiting AEs (defined in **1.1.5**).
7. Evidence of pregnancy.
8. Clinically significant changes from Baseline in ECG considered treatment-related including an increase from Baseline to an absolute heart rate corrected QT interval using Fridericia's formula (QTcF) value of ≥500 milliseconds (msec). Single ECGs will be repeated at 5 minute intervals 3 times to confirm any increased QT interval values. In the event of increased QTcF, the participant will be monitored until the value returns to below 500 msec.
9. Severe laboratory test abnormalities (defined in **1.1.6**).
10. Voluntary withdrawal. The participant (or participant's legally acceptable representative) wishes to withdraw from the study.

If a subject is prematurely withdrawn from the study drug, the Investigator will make every effort to perform the Early Termination visit (in Study Centre) and a further Follow-up contact 7-14 days after the last dose of study drug. Subjects who discontinue due to a Treatment-emergent adverse events (TEAEs) will be followed with appropriate medical management until there is a return to normal/Baseline values or a clinical diagnosis of an emergent illness is confirmed.

1.1.4 Replacement of Subjects

Participants who withdraw due to AEs which may be attributed to study treatment will not be replaced. Participants who withdraw due to personal reasons, protocol deviations or are lost to follow-up may be replaced until approximately 24 evaluable participants in Part A have completed the first 12 weeks of dosing.

1.1.5 Dose De-escalation

Dosing will continue unless dose limiting AEs or lack of tolerability are observed. If dose limiting AEs are observed at 100 mg, subsequent subjects with comparable characteristics may be dosed with a lower dose. Subjects not experiencing dose limiting AEs or a lack of tolerability will continue on their starting dose level i.e., 100 mg dose. Due to the genetic heterogeneity of mitochondrial disease, it is possible that some individuals could tolerate a higher dose level than others.

A dose limiting AE is defined as:

1. A serious AE (SAE) related to study drug, or
2. A SAE related to study drug in more than one subject, or
3. A pattern of clinically significant AEs related to study drug of moderate severity noted in ≥2 subjects
4. A clinically significant elevation (≥5 x ULN) in CK related to study drug and is accompanied by evidence of acute kidney injury (e.g., myoglobinuria).

If two subjects experience a clinically relevant moderate or greater severity AE of the same pathophysiology/System Organ Class (SOC) or Medical Dictionary for Regulatory Activities (MedDRA) preferred term that are related to study drug, the Chief Investigator (CI), Medical Monitor and Sponsor will determine whether these events would meet the definition for a dose limiting AE. In addition, events that are considered related to the study drug of any grade, may, in the judgment of the CI and Medical Monitor be categorized as a dose limiting AEs if they are deemed serious or sufficiently severe to preclude continued dosing of that subject.

AEs will be assessed for severity by the Investigator according to the definition below:

- Mild: An AE that does not interfere with usual activities;
- Moderate: An AE that interferes with usual activities;
- Severe: An AE that prevents usual activities

All clinical and laboratory data will be used to determine whether to continue to dose or to de-escalate dose. If no subject experiences a dose limiting AE, dosing will continue. If a dose limiting AE occurs, enrolment into the study will be put on hold, and the data will be reviewed within 3 days by the CI, Medical Monitor and the Sponsor to determine if lower dose levels should be tested in subjects with comparable disease characteristics to those who experienced the dose limiting AEs.

1.1.6 Withdrawal due to Laboratory Test Abnormalities

It is anticipated that subjects with mitochondrial myopathies will present with transient and non-clinically significant elevations in CK. In addition to the standard monitoring of clinical safety parameters, the following guidelines are provided for the monitoring of CK based on preclinical and clinical observations.

Confirmation Guidance: At any time during the study, the initial clinical laboratory results meeting the safety monitoring criteria (below) will be confirmed by performing measurements on new specimens which will take place as soon as possible and no later than 3 days of the initial laboratory test.

Stopping Rule Guidance: If any of the stopping criteria described below are met and confirmed, discontinuation of subject dosing will be considered. If a decision is made to permanently discontinue a subject, the subject will be evaluated and encouraged to complete the Early Termination procedures. In general, subjects who do not meet the stopping rules based on retest may continue dosing. However, the Investigator, Sponsor and Medical Monitor will confer as to whether additional close monitoring is appropriate.

Safety Monitoring for Serum Creatine Kinase

Subjects with PMM often present with mild to moderate Baseline elevations in CK and can experience transient episodes of significantly elevated CK induced by vigorous exercise, illness, or metabolic stress. The CK elevations are generally of the skeletal muscle fraction. However, a small proportion of the total CK may be from the myocardial fraction reflecting a small amount found in skeletal muscle rather than the presence of myocardial disease. Elevations in serum aminotransferase (ALT) and uric acid can accompany CK elevations and may not be indicative of liver disease. Not all individuals with elevated CK present with clinically significant rhabdomyolysis or myoglobinuria. Acute kidney injury is a complication of rhabdomyolysis. The risk of acute kidney injury is low in subjects with CK levels less than 15,000 international units per litre (IU/L).

1. In a subject with a normal CK at Baseline has a blood CK level >5 x ULN or if a subject with a 2-3 x ULN CK at Baseline has blood CK level ≥5 x Baseline level, she/he will be promptly contacted and evaluated for the presence of muscle pain, stiffness, cramping and myoglobinuria. If no clinical symptoms and no urine colour change (i.e., reddish-brown urine) is present, the subject will be retested with a urine dipstick (for signs of myoglobinuria) and for CK levels daily until levels stabilize or begin to recover. Subjects may continue to receive study drug. If myoglobinuria is present or CK levels have not stabilized or recovered after 7 days, the Investigator, Sponsor and Medical Monitor will discuss discontinuation of study drug.
2. If urine colour change indicates possible myoglobinuria (i.e., reddish-brown urine), study drug will be suspended, and the subject will promptly be admitted to the hospital for appropriate management of myoglobinuria.

If increases in CK are not related to study drug, a reduction in study dose level will not be required and affected subjects may be able to re-start study treatment if agreed by the Investigator, Medical Monitor and Sponsor.

Management of Liver Chemistry Elevations

Subjects will be withdrawn from the study if they have abnormal laboratory results that represent a significant danger to the subject as assessed by the Investigator. This decision will be made in the best interest of the subject with follow up using standard clinical practices. In the event of abnormalities considered clinically significant but not posing a significant danger to the subject, continuation of the study drug may be considered after discussion with the Medical Monitor and Sponsor. If the following changes in liver-related laboratory tests occur at any time during the study

- ALT or Aspartate aminotransferase (AST) >8 x ULN, or
- ALT or AST >5 x ULN for >2 weeks, or
- ALT or AST >3 x ULN in conjunction with elevated total bilirubin (TB) >2 x ULN or international normalized ratio (INR) >1.5 without initial findings of cholestasis (elevated alkaline phosphatase (AP)) AND no other reason found to explain the combination of increased AST/ALT and TB, such as viral hepatitis A, B, or C; pre-existing or acute liver disease, or another drug capable of causing the observed injury, or ALT or AST >3 x ULN with appearance of fatigue, nausea, vomiting, right upper quadrant pain or tenderness, fever, rash and/or eosinophilia (>5%).

Study medication will be discontinued immediately, and appropriate clinical care provided including an evaluation for other causes of liver injury with repeat laboratory tests, HBsAg, HBcAb, antibody to hepatitis A virus (anti-HAV), immunoglobulin M and G, AST, ALT, AP (including isoenzymes), TB (direct and indirect), one stage prothrombin time, serum proteins and, if clinically warranted, cytomegalovirus, toxoplasma and heterophile antibody titres. Appropriate medical care and follow up will be continued until the subject's laboratory profile has returned to normal/Baseline status.

If at any point the ALT or AST is elevated >1.5 x ULN, laboratory tests will be repeated ideally within 48-72 hours and no later than 7 days. If the ALT or AST remain elevated >1.5 x ULN and the observation cannot be explained by concomitant disease or another alternative aetiology, the abnormality will be recorded as an AE. The Investigator will contact the Medical Monitor for consideration of discontinuation of study medication, and discussion of possible alternative aetiologias. Subjects who have laboratory results out of range, but not deemed clinically significant by the Investigator can continue in the study.

1.1.7 Temporary Discontinuations

If a subject misses one or more consecutive doses due to personal circumstances or due to an AE (not related to study drug), they will be allowed to restart dosing after discussion between the Investigator, Medical Monitor and Sponsor.

Subjects may also be temporarily discontinued from study drug while AEs and/or laboratory test abnormalities are being investigated and/or at the request of the Medical Monitor. The Investigator will discuss with the Medical Monitor and Sponsor any subject for whom temporary or permanent discontinuation of study drug is being considered.

1.1.8 Review of Safety Data

Review of subject safety data (AEs, ECGs, laboratory safety tests, vital signs, and ophthalmic examinations) will be performed by The Sponsor (Reneo Pharmaceuticals, Inc.) Safety Review Team throughout the study in accordance with a study safety monitoring plan in order to identify any potential safety signals.

1.1.9 Adjustment of Dose

No dose adjustment will be conducted for any individual subject who may exhibit intolerability to the dose level tested. The overall study dose may be modified to a lower dose for subsequent subjects after a safety review made by the CI, the Medical Monitor, and the Sponsor.

1.2 Statistical Methods

1.2.1 Sample Size

It is envisaged that ~30 subjects will be screened to achieve 24 mavodelpar (REN001) dosed subjects in Part A. The sample size was selected without statistical considerations; however, it is considered adequate to meet the study objectives.

1.2.2 Statistical Analysis Plan

No formal statistical testing is planned. All endpoints will be summarized overall using both intention to treat (ITT) population and a completers analysis population (subgroup of participants who completed the 12 week and/or 48 Week trial endpoints). If the study drug dose is reduced during the study, data may also be summarized by dose. For all outcome measures, changes from Baseline at Week 12 will be summarized using descriptive statistics for continuous data (e.g., mean, SD, 95% confidence interval) and frequency tables (counts and percentages) to summarize improvements, no change or worsening for categorical data. For subjects who complete Part B, changes from baseline at Week 48 will also be summarized for outcome assessments performed.

Demographics, Baseline characteristics, and concomitant medication data will be summarized. TEAEs will be reported according to the System Organ Class (SOC) and preferred term, using the Medical Dictionary for Regulatory Activities (MedDRA), version 11.0 coding dictionary (https://www.meddra.org/). MedDRA coding dictionaries will also be used for the concomitant medications and medical histories. Baseline will be taken as the last measurement prior to dosing. No imputation of missing data for early terminations will be performed. The safety set used for safety analyses will include all subjects who received at least one dose of study treatment.

1.2.3 Disposition of Subjects

The number and percentage of subjects entering and completing each part of the study will be presented. Reasons for withdrawal will also be summarized.

1.2.4 Analysis Populations

- ***Full Analysis Set (FAS) and Safety Set:*** All subjects who received ≥1 dose of study treatment.
- ***Completers Analysis Set:*** Subgroup of participants who completed the 12 week trial endpoints.
- ***PK Set:*** All subjects in the FAS who receive study drug and have ≥1 evaluable post-dose pharmacokinetic (PK) measurement (even if this is <Level of Quantification (LOQ).
- ***Pharmacodynamic Set:*** All subjects in the FAS who receive study drug and have ≥1 evaluable post-dose Pharmacodynamic (PD) measurement (even if this is <LOQ).

Safety analyses will be based on the Safety Set. The FAS will be used in the analyses of all outcome measures. After data has been verified/coded/entered into the database, a review will be performed to define the analysis populations and check the quality of the data, identify outliers, and make decisions on how to deal with any data issues (e.g., missing values, withdrawals, protocol deviations). After the pre-analysis review, resolution of all issues and documentation of all decisions, the database will be locked.

1.2.5 Safety Analysis

Treatment-emergent adverse events (TEAEs) will be summarized by SOC and preferred term, using the MedDRA, version 11.0 (https://www.meddra.org/) coding dictionary. The number of subjects reporting each AE preferred term will be tabulated for all TEAEs and separately for those considered as possibly or probably related to study treatment by the Investigator. Number of subjects reporting SAEs will also be tabulated. Changes from Baseline in laboratory parameters, vital signs and ECG data will be summarized by visit. Changes of potential clinical significance will be flagged and summarized, such as CK values (e.g., >5 x ULN) and ECG values (e.g., increase in QTcF ≥30 msec from Baseline or an absolute QTcF value of ≥500 msec.)

1.2.6 Pharmacokinetic Endpoints

Pharmacokinetics (PK) of mavodelpar (REN001) was investigated using population PK methodology;[13] mavodelpar plasma concentrations will be summarized over time using descriptive statistics. Individual concentration-time data on Day 1 and Week 12 will be plotted over PK sampling times. Median profiles of concentration-time data, using nominal PK sampling times, will be presented as well as linear-linear, linear-log plots.

Supplementary References (Study Protocol Summary)

1 Mancuso M, McFarland R, Klopstock T, Hirano M, consortium on Trial Readiness in
Mitochondrial M. International Workshop:: Outcome measures and clinical trial
readiness in primary mitochondrial myopathies in children and adults. Consensus
recommendations. 1618 November 2016, Rome, Italy. Neuromuscul Disord. 2017
2 Dec;27(12):1126-37.
Levey AS, Stevens LA, Schmid CH, Zhang YL, Castro AF, 3rd, Feldman HI, et al. A new
3 equation to estimate glomerular filtration rate. Ann Intern Med. 2009 May
5;150(9):604-12.
4 Engel WK, Cunningham GG. Rapid Examination Of Muscle Tissue. An improved
trichrome method for fresh frozen biopsy sections. Neurology. 1963 Nov;13:919-23.
5 Jones CJ, Rikli RE, Beam WC. A 30-s chair-stand test as a measure of lower body
strength in community-residing older adults. Res Q Exerc Sport. 1999 Jun;70(2):113-
9.
6 Schaefer AM, Phoenix C, Elson JL, McFarland R, Chinnery PF, Turnbull DM.
Mitochondrial disease in adults: a scale to monitor progression and treatment.
7 Neurology. 2006 Jun 27;66(12):1932-4.
Corporation R. RAND 36-Item Health Survey (Version 1.0). [cited 08.06.2021];
Available from: https://www.rand.org/health-care/surveys_tools/mos/36-item-
8 short-form/scoring.html
Fisk JD, Ritvo PG, Ross L, Haase DA, Marrie TJ, Schlech WF. Measuring the functional
impact of fatigue: initial validation of the fatigue impact scale. Clin Infect Dis. 1994
9 Jan;18 Suppl 1:S79- 83.
Blakely E, He L, Gardner JL, Hudson G, Walter J, Hughes I, et al. Novel mutations in the
TK2 gene associated with fatal mitochondrial DNA depletion myopathy. Neuromuscul
10 Disord. 2008 Jul;18(7):557-60.
White HE, Durston VJ, Seller A, Fratter C, Harvey JF, Cross NC. Accurate detection and
quantitation of heteroplasmic mitochondrial point mutations by pyrosequencing.
Genet Test. 2005 Fall;9(3):190-9.
11 Rocha MC, Grady JP, Grunewald A, Vincent A, Dobson PF, Taylor RW, et al. A novel
immunofluorescent assay to investigate oxidative phosphorylation deficiency in
mitochondrial myopathy: understanding mechanisms and improving diagnosis. Sci
12 Rep. 2015 Oct 15;5:15037.
Porteous WK, James AM, Sheard PW, Porteous CM, Packer MA, Hyslop SJ, et al.
Bioenergetic consequences of accumulating the common 4977-bp mitochondrial DNA
13 deletion. Eur J Biochem. 1998 Oct 1;257(1):192-201.
Strehler BL. Adenosine-5'-triphosphate and Creatine Phosphate Determination with
Luciferase. In: Bergmeyer HU, ed. *Methods of Enzymatic Analysis (Second Edition)*:
Academic Press 1974:2112-26.
Williams PJ, Ette EI. The role of population pharmacokinetics in drug development in
light of the Food and Drug Administration's 'Guidance for Industry: population
pharmacokinetics'. Clin Pharmacokinet. 2000 Dec;39(6):385-95
